# Effectiveness and safety of self-management interventions for improving glycemic control and health-related quality of life among adults with type 2 diabetes mellitus in sub-Saharan Africa: a systematic review and meta-analysis

**DOI:** 10.11124/JBIES-23-00273

**Published:** 2024-06-24

**Authors:** Naomi Carter, Gamze Nalbant, Prit Chahal, Kaushik Chattopadhyay

**Affiliations:** 1 Lifespan and Population Health, School of Medicine, University of Nottingham, Nottingham, United Kingdom; 2 Health Education England, East Midlands, Leicester, United Kingdom; 3 The Nottingham Centre for Evidence-Based Healthcare: A JBI Centre of Excellence, The University of Nottingham, Nottingham, United Kingdom

**Keywords:** meta-analysis, self-management, sub-Saharan Africa, systematic review, type 2 diabetes mellitus

## Abstract

**Objective:**

The objective of this review was to assess and synthesize evidence on the effectiveness and safety of self-management interventions for improving glycemic control and health-related quality of life among adults with type 2 diabetes mellitus (T2DM) in sub-Saharan Africa.

**Introduction:**

There has been a rapid increase in the prevalence of T2DM in sub-Saharan Africa. Lifestyle-related risk factors require self-management strategies, and these must be tailored to the context. Several randomized controlled trials (RCTs) evaluating T2DM self-management interventions in sub-Saharan Africa have been conducted.

**Inclusion criteria:**

This systematic review included RCTs assessing the effectiveness and safety of self-management interventions among adults with T2DM in sub-Saharan Africa, where the self-management intervention matched at least 1 category of the Practical Reviews in Self-Management Support (PRISMS) for long-term conditions taxonomy.

**Methods:**

The following databases were searched from inception until January 14, 2023: MEDLINE (Ovid), PubMed, Embase (Ovid), CINAHL (EBSCOhost), PsycINFO (Ovid), Scopus, Cochrane Central Register of Controlled Trials (CENTRAL), Directory of Open Access Journals, EThOS, and ProQuest Dissertations and Theses (ProQuest). Global Health (EBSCOhost) was searched from inception until June 8, 2021. OpenGrey was searched from inception until its archive date of December 1, 2020. Two independent reviewers conducted title and abstract screening, full-text screening, data extraction, and critical appraisal. Disagreements were resolved through discussion or with a third reviewer. Data synthesis was conducted narratively, followed by meta-analysis where feasible. The Grading of Recommendations, Assessment, Development and Evaluation (GRADE) approach for assessing the certainty of evidence was applied.

**Results:**

From 2699 records identified, 18 RCTs were included in the systematic review and 14 in the meta-analysis. Interventions included broad self-management education programs, peer support, exercise interventions with education, nutrition education, educational text messaging, and blood glucose self-monitoring support. Only 4 studies received a “yes” response for more than half of the criteria in the standardized JBI critical appraisal tool for RCTs. Compared to the control, self-management interventions did not significantly reduce glycated hemoglobin (HbA1c) levels at 3 months (302 participants, mean difference [MD] –6.0 mmol/mol, 95% CI –17.5, 5.4; very low certainty on GRADE assessment) or 12 months (1504 participants, MD –3.7 mmol/mol, 95% CI –8.2, 0.7; moderate certainty on GRADE assessment). HbA1c was significantly reduced at 6 months (671 participants, MD –8.1 mmol/mol, 95% CI –10.7, –5.4; low certainty on GRADE assessment). Four studies assessed health-related quality of life, but only 1 demonstrated an improvement (2205 participants). Three studies reported no adverse events in relation to the trial interventions (1217 participants), and adverse events were not reported in the remainder of studies. There did not appear to be clinically significant effects on body mass index, lipid profile, or systolic or diastolic blood pressure. The evidence was mixed for weight and waist circumference.

**Conclusions:**

Self-management interventions for adults living with T2DM in sub-Saharan Africa may produce a clinically significant improvement in glycemic control at 6 months, but this may wane in the long term. There was not convincing evidence to indicate a benefit of these interventions on health-related quality of life, but reporting on this outcome measure was limited. There were insufficient data on adverse events to be able to draw conclusions.

**Review registration:**

PROSPERO CRD42021237506

## Summary of findings

**Table:** 

Self-management interventions vs usual care, enhanced care, or sham intervention for improving glycemic control and health-related quality of life among adults with type 2 diabetes mellitus in sub-Saharan Africa					
Bibliography: Carter N, Nalbant G, Chahal P, Chattopadhyay K. Effectiveness and safety of self-management interventions for improving glycemic control and health-related quality of life among adults with type 2 diabetes mellitus in sub-Saharan Africa: a systematic review and meta-analysis. JBI Evid Synth. 2024;22(9):1715-88.					
Interactive Summary of Findings [iSoF] table: https://gdt.gradepro.org/presentations/#/isof/isof_1b4993c5-f3eb-4da8-bc92-0ea08e150a7e-1707089448024					
Outcomes	Anticipated absolute effects (95% CI)		No of participants (studies)	Certainty of the evidence (GRADE)	Comments
	Risk with usual care, enhanced usual care, or sham intervention	Risk with self-management interventions			
HbA1c at 3 months (measured using venous blood sampling)	The mean HbA1c ranged from 51-81 mmol/mol	MD 6.03 mmol/mol lower (17.46 lower to 5.40 higher)	302 (4 RCTs)	⊕○○○ Very low^a,b,c^	No significant effect of self-management interventions on HbA1c at 3 months compared to control was found, but the evidence was very uncertain.
HbA1c at 6 months (measured using venous blood sampling)	The mean HbA1c ranged from 64-91 mmol/mol	MD 8.06 mmol/mol lower (10.67 lower to 5.44 lower)	671 (7 RCTs)	⊕⊕○○ Low^a,d^	Self-management interventions may result in a significant reduction in HbA1c at 6 months compared to control.
HbA1c at 12 months (measured using venous blood sampling)	The mean HbA1c ranged from 81-92 mmol/mol	MD 3.74 mmol/mol lower (8.18 lower to 0.69 higher)	1504 (6 RCTs)	⊕⊕⊕○ Moderate^a^	Self-management interventions probably have no significant effect on HbA1c at 12 months compared to control.
Fasting blood glucose at 6 months (measured using venous or fingerprick blood sampling)	The mean fasting blood glucose was 10.50 mmol/L	MD 1.76 mmol/L lower (2.74 lower to 0.78 lower)	202 (2 RCTs)	⊕○○○ Very low^a,c,d^	A significant reduction in fasting blood glucose at 6 months with self-management interventions compared to control was found, but the evidence was very uncertain.
HRQoL at 4 to 12 months (measured using any generic or disease-specific standardized questionnaire)	Three studies found no significant effect of self-management interventions on HRQoL compared to the control, while one did find a significant improvement for all HRQoL domains, apart from pain. The combined findings do not appear to support a significant effect of self-management interventions on HRQoL outcomes from the limited available data.		2205 (4 RCTs)	⊕⊕○○ Low^a,e^	Self-management interventions may result in no significant difference in HRQoL at 4 to 12 months.
Adverse effects at 3 to 12 months (measured using any reported adverse event)	Three studies reported on adverse events, stating none occurred in relation to the trial interventions. There were insufficient data to confidently determine the effect of self-management interventions on the outcome of adverse events.		1217 (3 RCTs)	⊕⊕○○ Low^a,d^	Self-management interventions may have no significant effect on adverse events at 3 to 12 months, but there were insufficient data to confidently assess this.
HbA1c, glycated hemoglobin; HRQoL, health-related quality of life; MD, mean difference; RCT, randomized controlled trial.					
**GRADE Working Group grades of evidence.** **High certainty:** We are very confident that the true effect lies close to that of the estimate of the effect. **Moderate certainty:** We are moderately confident in the effect estimate: the true effect is likely to be close to the estimate of the effect, but there is a possibility that it is substantially different. **Low certainty:** Our confidence in the effect estimate is limited: the true effect may be substantially different from the estimate of the effect.					
**Explanations** a. lack of blinding in participants/outcome assessors. b. statistical heterogeneity. c. small sample size. d. risk of attrition bias. e. inconsistent findings among included studies.					

## Introduction

Type 2 diabetes mellitus (T2DM) is a chronic disease characterized by insulin resistance, insulin deficiency, and hyperglycemia.^1^ This form of diabetes accounts for approximately 90% of total diabetes cases globally.^2^ It is associated with a variety of complications that contribute to morbidity and mortality, including nephropathy, retinopathy, neuropathy, cardiovascular disease, and cerebrovascular disease.^3^ Managing T2DM with pharmacological treatment and lifestyle changes can reduce complications and improve the patient’s quality of life.^1,4^

The burden of T2DM is rapidly escalating globally as part of a wider pattern of increase in non-communicable diseases (NCDs).^5^ The impact of NCDs, such as T2DM, falls disproportionately on low- and middle-income countries (LMICs), which account for 86% of NCD-related premature deaths.^6^ Of all worldwide regions, sub-Saharan Africa is predicted to have the greatest proportional increase in the number of adults living with diabetes, from approximately 23.6 million in 2021 to 55.2 million in 2045.^7^ This is partly driven by population growth, but also a change in lifestyle factors that affect T2DM risk, such as diet and physical activity levels.^8^ This will have profound consequences on morbidity and mortality, reflected in rising associated disability-adjusted life years.^8^ There are also significant cost implications. Diabetes-related health expenditure for the region is expected to triple between 2021 and 2045, despite significant resource constraints in many sub-Saharan African settings.^7^

Self-management interventions are any structured intervention aimed at improving self-care behaviors in people with chronic diseases, for which the format and content can vary.^9^ The scope of these interventions is defined in the taxonomy proposed in the Practical Systematic Review of Self-Management Support (PRISMS) for long-term conditions, which provides 14 categories.^9^ These include: i) education about condition and management, ii) information about available resources, iii) provision of/agreement on specific action plans and/or rescue medication, iv) regular clinical review, v) monitoring of condition with feedback to the patient, vi) practical support with adherence (medication or behavioral), vii) provision of equipment, viii) safety netting, ix) training/rehearsal to communicate with health care providers, x) training/rehearsal for activities of daily living, xi) training/rehearsal for practical self-management activities, xii) training/rehearsal for psychological strategies, xiii) social support, and xiv) lifestyle advice and support. Self-management interventions for T2DM aim to build the confidence of those with the condition, improving their ability to undertake the wide variety of tasks that are required to optimize management and improve outcomes.^9^

Self-management interventions for T2DM are a core component of a number of T2DM guidelines.^1,10^ This is supported by high-level umbrella review evidence for their effectiveness in modestly reducing glycated hemoglobin (HbA1c) levels at 6 months, although this effect may wane over time.^11^ Despite including findings from 459 randomized controlled trials (RCTs) across 33 countries, no trials were included from sub-Saharan Africa.^11^ Self-management interventions are highly context-dependent and need to be tailored to the setting and culture in which they are applied.^12,13^ Context-specific assessment of their effectiveness is therefore required, as what works in one setting and culture cannot be presumed to work in other settings and cultures.^14^

Several RCTs evaluating T2DM self-management interventions in sub-Saharan Africa have been conducted, with variable effects demonstrated on glycemic control.^15–24^ A preliminary search was carried out on PubMed, Google Scholar, the Cochrane Database of Systematic Reviews, *JBI Evidence Synthesis*, and PROSPERO to identify any current or in-progress systematic reviews on the same or similar topics. Two pre-existing systematic reviews with some overlap in scope were identified.^25,26^ A variety of factors necessitated completion of the present review in addition to these reviews, including the need for a broader scope of self-management interventions for T2DM in the sub-Saharan African context; provision of T2DM-specific evidence rather than type 1 diabetes and T2DM combined; the need to include health-related quality of life (HRQoL) and safety outcomes as well as clinical outcomes; and the publication of further relevant RCTs since the previous reviews were conducted.^24,27–29^ Synthesizing safety outcomes in addition to glycemic control and HRQoL outcomes in non-pharmacological interventions is important in order to provide a balanced picture of their effectiveness. Examples of potential adverse events that could occur during self-management interventions include injury from exercise or hypoglycemia from dietary or exercise advice.^1^

This systematic review aimed to evaluate and synthesize evidence on the effectiveness and safety of self-management interventions for improving glycemic control and HRQoL among adults with T2DM in sub-Saharan Africa, to provide a comprehensive and up-to-date picture of the evidence base, and help inform local health policy and practice.

## Review questions

- Are self-management interventions effective for improving glycemic control among adults with T2DM in sub-Saharan Africa?
- Are self-management interventions effective for improving the HRQoL of adults with T2DM in sub-Saharan Africa?
- Are self-management interventions safe to use for adults with T2DM in sub-Saharan Africa?

## Inclusion criteria

### Participants

This systematic review included studies carried out among adults (≥18 years) with T2DM living in sub-Saharan Africa, as defined by the World Bank.^30^ This included the following 48 countries: Angola, Benin, Botswana, Burkina Faso, Burundi, Cabo Verde, Cameroon, Central African Republic, Chad, Comoros, Côte d’Ivoire, Democratic Republic of Congo, Equatorial Guinea, Eritrea, Eswatini, Ethiopia, Gabon, The Gambia, Ghana, Guinea, Guinea-Bissau, Kenya, Lesotho, Liberia, Madagascar, Malawi, Mali, Mauritania, Mauritius, Mozambique, Namibia, Niger, Nigeria, Republic of Congo, Rwanda, São Tomé and Principe, Senegal, Seychelles, Sierra Leone, Somalia, South Africa, South Sudan, Sudan, Tanzania, Togo, Uganda, Zambia, and Zimbabwe.

Where a study focused specifically on a diabetic comorbidity or complication and included relevant outcomes of interest, it was included if all participants had T2DM. Where participant age range was not specified, the study was included if the mean age of the participants was ≥18 years. Where studies included children, the study was eligible for inclusion if the mean age of the participants was ≥18 years or if the study findings were stratified into adults and children; however, this was not encountered. Studies that included participants with type 1 diabetes mellitus were excluded unless it was possible to extract the data on participants with T2DM only.

### Intervention

Studies were included if they assessed any self-management intervention for T2DM that matched at least 1 of the 14 categories of the PRISMS taxonomy.^9^ There were no limits regarding frequency, duration, or delivery mode of the intervention. Studies that assessed multiple self-management interventions were included. Studies that solely assessed supervised exercise interventions or dietary supplements were excluded, as these were not considered to represent a self-management intervention. Studies that assessed exercise interventions with additional components to support self-management (eg, through education or addressing barriers to exercise) were included.

### Comparator

Studies comparing self-management interventions with any or no intervention were included in this systematic review. Co-interventions were allowed if all the study arms received the same co-interventions. If a study included multiple arms, the authors included the arms that met the review inclusion criteria. Studies comparing 2 or more modes of delivery of the same self-management intervention without any other comparator were excluded.

### Outcomes

Studies that assessed any of the following primary outcomes of interest were included: HbA1c (mmol/mol measured using venous blood sampling), fasting blood glucose (FBG; mmol/L measured using venous or finger prick blood sampling), HRQoL (measured using any generic or disease-specific standardized questionnaire), and adverse effects (any reported adverse events). For those studies that reported on at least one of the primary outcomes, data on the following secondary outcomes of interest were also extracted where available: weight (kg), body mass index (BMI; kg/m^2^), waist circumference (WC; cm), systolic blood pressure (SBP; mmHg), diastolic blood pressure (DBP; mmHg), and lipid profile (total cholesterol, high-density lipoprotein [HDL] cholesterol, low-density lipoprotein [LDL] cholesterol, and/or triglycerides; mmol/L measured using fasting or non-fasting venous blood sample). These are standard components of monitoring cardiovascular risk in T2DM.^31^ Data were extracted at 3-month, 6-month, and 12-month time points from randomization. This was a deviation from the protocol, as we originally intended to extract outcome data at 6-month, 12-month, and 24-month time points; however, due to a lack of 24-month outcome data in the included studies, these time points were amended.

### Types of studies

Based on the hierarchy of study designs to assess effectiveness of interventions and the feasibility and practicality of the proposed work, only RCTs were included in this systematic review. Cluster RCTs were eligible for inclusion. For crossover RCTs, the first stage of the study prior to crossover was eligible for inclusion.

## Methods

This systematic review was conducted in accordance with JBI methodology for systematic reviews of effectiveness and followed a published, peer-reviewed, a priori protocol.^32,33^ The review was registered in PROSPERO (CRD42021237506).

### Search strategy

The search strategy was initially developed for MEDLINE (Ovid) using a combination of search terms and index terms in consultation with a senior research librarian at the University of Nottingham, UK. The T2DM component was based on the search strategies reported in the UK’s National Institute for Health and Care Excellence (NICE) guideline for managing T2DM and a relevant systematic review of effectiveness.^31,34^ The sub-Saharan Africa component was based on the World Bank list of country names and any known alternatives.^30^ The self-management interventions component was developed via an initial limited search in MEDLINE, with exploration of terms in the titles and abstracts of relevant papers, along with linked index terms. This was further developed using a previously published relevant search strategy.^9^ The study design component was based on the search strategy reported in the NICE guideline for managing T2DM.^31^ The search strategy was then adapted for the other listed information sources using the PolyGlot Search Translator, where possible, and in consultation with the senior research librarian.^35^ No language restrictions were applied.

The search strategy aimed to locate both published and unpublished studies via the following electronic databases and gray literature sources searched from inception until January 14, 2023: MEDLINE (Ovid), Embase (Ovid), CINAHL (EBSCOhost), PsycINFO (Ovid), Scopus, Cochrane Central Register of Controlled Trials (CENTRAL), Directory of Open Access Journals, EThOS, and ProQuest Dissertations and Theses (ProQuest). PubMed was also searched with the restriction “as supplied by publisher” to capture studies not yet indexed in MEDLINE.^36^ Global Health (EBSCOhost) was searched from inception until June 8, 2021, due to limited access. OpenGrey was searched from inception until its archive date of December 1, 2020. The reference lists of previous systematic reviews and all the studies included in the review were screened for additional studies. The full search strategies are provided in Appendix I.

### Study selection

Following the searches, all identified citations were collated and uploaded into EndNote v.X9 (Clarivate Analytics, PA, USA), and duplicates removed via manual screening assisted by the “Find duplicates” tool.^37^ The resulting citations were then transferred to Rayyan (Qatar Computing Research Institute, Doha, Qatar) for title and abstract screening.^38^ Titles and abstracts were screened by 2 independent reviewers (NC, GN) against the inclusion criteria for the systematic review. Studies identified as potentially eligible or those without an abstract had their full text retrieved and imported into Rayyan. The full texts were assessed in detail against the inclusion criteria by 2 independent reviewers (NC, GN). Any disagreements that arose between the reviewers during the study selection process were resolved through discussion or with a third reviewer (KC). Full-text studies that did not meet the inclusion criteria were excluded, and reasons for their exclusion are provided in Appendix II.

### Assessment of methodological quality

Included studies were critically appraised for methodological quality by 2 independent reviewers (NC, GN) using the standardized JBI critical appraisal tool for RCTs.^33^ The 2 reviewers independently answered each question and assigned a score as met (yes), not met (no), unclear, or not applicable. Any disagreements were resolved through discussion or with a third reviewer (KC). Regardless of their methodological quality, all included studies underwent data extraction and narrative synthesis, and were included in the meta-analysis, where possible.

### Data extraction

Data were extracted from included studies by 2 independent reviewers (NC, GN). Any disagreements that arose between the reviewers were resolved through discussion, thus a third reviewer was not required for this stage. A data extraction form was developed for the systematic review based on the standardized JBI data extraction tool incorporated within the JBI System for Unified Management, Assessment and Review of Information (JBI SUMARI; JBI, Adelaide, Australia), expanded with additional domains specific to this review.^39^ This data extraction form was piloted on 3 initial studies and then amended based on the discussion between 2 reviewers (NC, GN). Data extraction included study design and setting, participant characteristics, intervention and comparator details, outcomes of relevance to the review question and their timings, and whether the study was commercially funded. Post-intervention data (ie, scores in each group after the intervention) were preferred over change from baseline data (ie, post-intervention score minus the baseline score). Percentage change from the baseline was not included.^40^

For randomized crossover study designs, only data from the first stage prior to crossover were included in the review. Where the intra-cluster correlation coefficient was not available from the cluster RCTs, the findings from these studies could not be pooled with those from individually randomized study designs. Their findings were, therefore, presented and synthesized narratively alongside the relevant meta-analyses.

In the case of missing data or where clarification was required, corresponding authors of the included studies were contacted by email 2 times. If still unavailable, for example, in the instance of a missing SD, it was estimated based on equations using the standard error or 95% CI.^41^ If only median and IQRs were reported, mean was assumed to be equal to median, and the SD was calculated (IQR/1.35).^42^

### Data synthesis

Studies were synthesized narratively and, where possible, pooled with statistical meta-analysis using the inverse variance statistical method (where study weight is determined by the precision of the effect estimate), and based on a random-effects model, to provide a weighted measure of intervention effect using JBI SUMARI.^33,39,43^ Syntheses were planned for each of the primary and secondary outcomes at each of the prespecified time points, with included studies allocated to each synthesis according to the availability of data from that study for each outcome at each time point.

For continuous outcomes, where all studies used the same scale or the scales could be converted to a single standard unit, weighted mean difference (MD) with 95% CI were reported. The preferred unit for each outcome is listed under the Outcomes section. HbA1c unit conversions were done using online calculators.^44,45^ For the categorical outcome of adverse events, it was planned to present risk ratios with 95% CI.

Where a study had multiple arms, the comparisons between the intervention and control arms were included in separate meta-analysis models to avoid the issue of double counting. Where possible, analyses were based on the intention-to-treat (ITT) principle.

Clinical and methodological heterogeneity (diversity) were assessed by descriptively comparing trial and participant characteristics between the studies. The authors quantified heterogeneity using the *I* ^2^ statistic and categorized heterogeneity as substantial where values were greater than 50%.

Subgroup analyses were conducted where a significant effect on a primary outcome measure was found and where there were sufficient data to investigate, using RevMan v.5.4.1 (Copenhagen: The Nordic Cochrane Centre, Cochrane). This was planned for group vs individual self-management intervention design, primary category of self-management intervention according to the PRISMS taxonomy, face-to-face vs remote delivery method, intervention delivery by professional vs lay person, and comparator of no intervention vs any intervention. Where a significant effect on a primary outcome measure was found, sensitivity analyses were planned to assess the robustness of the results by excluding studies that were of poor methodological quality (ie, no or unclear scores assigned to allocation concealment, blinding of outcome assessor, and ITT analysis on the standardized JBI critical appraisal tool for RCTs^33^); commercially funded (full, partial, or unclear); not written in English; or not a journal publication (ie, not peer-reviewed).

### Assessing certainty in the findings

The Grading of Recommendations, Assessment, Development and Evaluation (GRADE) approach for grading the certainty of evidence was followed,^46^ and a Summary of Findings was created using GRADEpro GDT (McMaster University, ON, Canada). This was undertaken by 2 independent reviewers (NC, GN) at the outcome level. The findings were initially ranked as high and were downgraded to moderate, low, or very low if there was evidence of the following: risk of bias, inconsistency of results, imprecision, and/or publication bias. Indirectness of evidence was not anticipated in light of the specificity of the inclusion criteria. In the risk of bias domain, the following were considered for downgrading: no allocation concealment (“no” to Q2 in the JBI critical appraisal checklist for RCTs), lack of blinding (“no” to Q4 or Q6), and attrition bias (“no” to Q9).^34^ If one of these 3 were present in the majority of studies (ie, >50%), then it was downgraded by 1 level. If more than one issue were present, then it was downgraded by 2 levels. In the inconsistency of results domain, if the statistical heterogeneity (ie, *I* ^2^ statistic) was >50%, then it was downgraded by 1 level, and if ≥90%, then it was downgraded by 2 levels. Where meta-analysis was not possible for an outcome, the authors assessed for conflicting results among included studies and made a judgment to downgrade by 1 or 2 levels depending on the degree of unexplained conflict. In the imprecision domain, if the total sample size was 100 to <400 then it was downgraded by 1 level, and if <100 then by 2 levels. It was planned to assess for publication bias with a funnel plot where the number of studies included in a single meta-analysis was 10 or more, and to downgrade by 1 level if publication bias was present. As no meta-analyses had 10 or more studies, this was not required.

## Results

### Study inclusion

**Figure 1:**
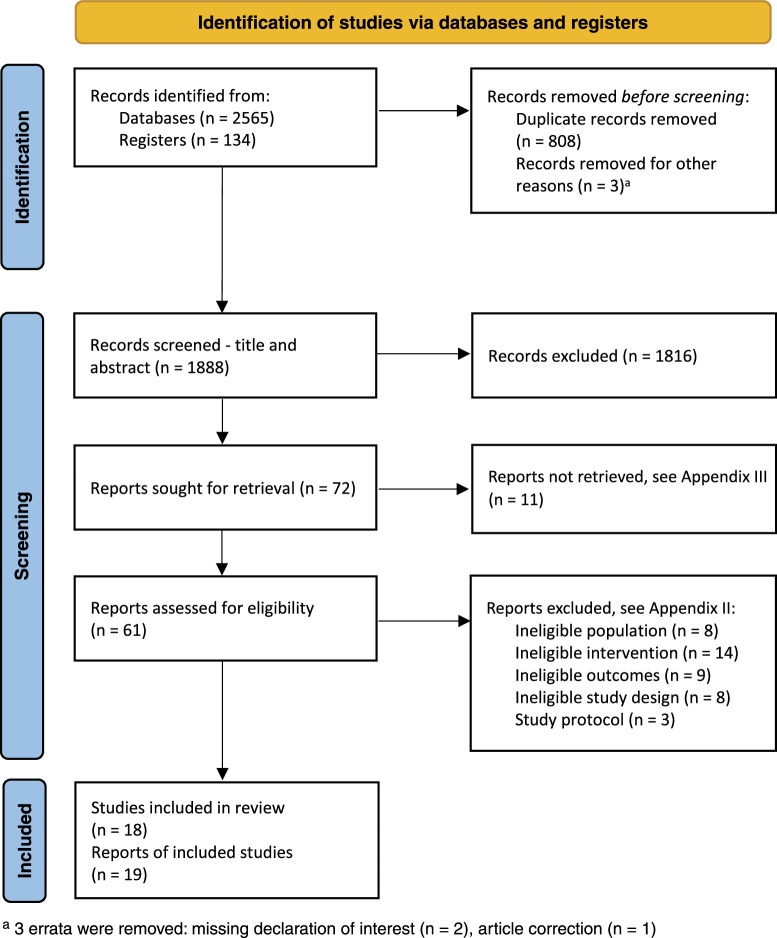
Search results and study selection and inclusion process^47^

The study selection process is summarized in a Preferred Reporting Items for Systematic Reviews and Meta-Analyses (PRISMA) flow diagram (Figure 1).^47^ A total of 2699 records were retrieved from the searches, including 2565 from databases and 134 from registers. Following removal of duplicates, 1888 records underwent title and abstract screening, of which 72 reports were sought for full-text review. Eleven reports were not retrieved (reasons provided in Appendix III). Of the 61 full-text reports assessed for eligibility, 19 reports from 18 studies met the inclusion criteria.^17,18,20–24,27–29,48–56^ There were 2 reports from a single study.^50,51^ Eight reports were excluded due to ineligible study populations, 14 due to ineligible interventions, 9 due to ineligible outcome measures, 8 due to ineligible study design, and 3 as protocols with no corresponding full text or available data (Appendix II). A review of the reference lists of included studies did not provide any additional reports.

### Methodological quality

Limitations of methodological quality among the included studies were apparent, as only 4 were assigned a “yes” for more than half of the criteria in the standardized JBI critical appraisal tool for RCTs (Table 1).^20,23,28,48^ Fourteen studies reported true randomization,^18,20–23,27–29,48–52,54,55^ while randomization procedures were unclear for the remaining 4.^17,24,53,56^ Explicit and clear reporting of allocation concealment was present in 10 studies,^20,21,23,24,27,28,48,50,51,54,55^ while for the remainder, this was unclear due to not being adequately described.^17,18,22,29,49,52,53,56^ Only 2 studies reported that participants were blinded to treatment assignment.^20,49^ The nature of self-management interventions can make blinding participants to treatment assignment challenging; however, this can be achieved (eg, through use of sham interventions).^20^ As most participants had knowledge of their group assignment, there was a risk of response bias in favor of finding an intervention effect. Bias in this direction may also have been compounded by a low rate of blinding of outcome assessors (6 studies).^27,28,48,50,51,54,55^

**Table 1:** Critical appraisal of included randomized controlled trials

Study	Q1	Q2	Q3	Q4	Q5	Q6	Q7	Q8	Q9	Q10	Q11	Q12	Q13
Asante *et al.*, 2020^27^	Y	Y	N	N	N	Y	U	Y	Y	U	U	U	Y
David *et al.*, 2021^49^	Y	U	U	Y	N	N	U	U	U	Y	U	Y	Y
Debussche *et al.*, 2018^18^	Y	U	N	N	N	U	U	N	N	Y	Y	Y	Y
Farmer *et al.*, 2021^28^	Y	Y	Y	U	Y	Y	U	Y	Y	U	Y	Y	Y
Fayehun *et al.*, 2018^20^	Y	Y	Y	Y	N	N	U	Y	Y	Y	U	Y	Y
Gathu *et al.*, 2018^21^	Y	Y	N	N	N	U	U	N	N	U	Y	Y	Y
Hailu *et al.*, 2018^50^ and 2021^51^	Y	Y	N	U	N	Y	U	N	N	U	U	N	Y
Mash *et al.*, 2014^22^	Y	U	N	N	N	N	U	Y	Y	U	Y	Y	Y
Muchiri *et al.*, 2016^23^	Y	Y	Y	N	N	U	U	Y	Y	U	Y	Y	Y
Muchiri *et al.*, 2021^48^	Y	Y	Y	N	N	Y	U	N	Y	U	Y	Y	Y
Ng’ang’a *et al.*, 2022^52^	Y	U	N	U	N	U	U	N	N	U	U	Y	Y
Ojieabu *et al.*, 2017^17^	U	U	Y	U	N	U	U	U	U	U	U	N	Y
Ojieabu, 2020^53^	U	U	N	U	N	U	U	U	U	U	U	U	Y
Pienaar *et al.*, 2021^29^	Y	U	N	N	N	U	U	N	N	U	Y	N	U
Thuita *et al.*, 2020^24^	U	Y	N	U	N	U	U	Y	N	Y	Y	Y	Y
van Rooijen *et al.*, 2004^54^	Y	Y	Y	N	N	Y	U	N	N	U	U	Y	Y
van Rooijen *et al.*, 2010^55^	Y	Y	N	U	N	Y	U	N	N	U	U	Y	Y
Wargny *et al.*, 2018^56^	U	U	N	N	N	U	U	Y	N	U	Y	Y	U
Total %	78	56	33	11	6	33	0	39	33	22	50	72	89

Y, yes; N, no; U, unclear.

JBI critical appraisal checklist for randomized controlled trials.

Q1: Was true randomization used for assignment of participants to treatment groups?

Q2: Was allocation to treatment groups concealed?

Q3: Were treatment groups similar at baseline?

Q4: Were participants blind to treatment assignment?

Q5: Were those delivering treatment blind to treatment assignment?

Q6: Were outcome assessors blind to treatment assignment?

Q7: Were treatment groups treated identically other than the intervention of interest?

Q8: Was follow-up complete, and if not, were strategies to address incomplete follow-up utilized?

Q9: Were participants analyzed in the groups to which they were randomized?

Q10: Were outcomes measured in the same way for treatment groups?

Q11: Were outcomes measured in a reliable way?

Q12: Was appropriate statistical analysis used?

Q13: Was the trial design appropriate, and any deviations from the standard RCT design (individual randomization, parallel groups) accounted for in the conduct and analysis of the trial?

The nature of the self-management interventions assessed in the included studies appeared to have precluded blinding of those delivering treatment to treatment assignment, with the exception of a mass SMS text messaging intervention for which blinding of those delivering treatment was achieved.^28^ The absence of blinding may have increased the risk of performance bias. The review authors decided that, in order to determine that treatment groups were treated identically other than the intervention of interest, a specific statement to this effect was required. This was not present in any of the studies, resulting in unclear scores throughout for this criterion. Specific description of outcomes being measured in the same way for treatment groups was also required for a response of “yes” for question 10, which was only present in 4 studies.^18,20,24,49^ While this may have been the case for the remainder of the studies, it would have to be inferred and was therefore marked as “unclear.”

Additional risk of bias from the included studies comes from broadly low rates of ITT analysis (6 studies),^20,22,23,27,28,48^ and analysis of the potential impact of loss to follow-up (7 studies).^20,22–24,27,28,56^ Steps to ensure reliability of outcome measurement were explicit in half of the included studies^18,21–24,28,29,48,56^ and unclear in the remainder.^17,20,27,49–55^ Most included studies used appropriate statistical analysis, including power calculations.^17,18,20–24,27,28,48–55^ For one cluster RCT and one crossover study, it was unclear whether the clustered nature of the study groups had been adequately accounted for in analysis.^29,56^

### Characteristics of included studies

Characteristics of included studies are presented in Appendix IV. Of the 18 studies, 15 were individually randomized,^17,18,20,21,23,24,27,28,48–55^ 2 were cluster randomized,^22,29^ and 1 had a cluster randomized crossover design.^56^ The included cluster RCTs and crossover study are presented and synthesized narratively alongside the relevant meta-analyses, as the intra-cluster correlation coefficient was not available to allow pooling. One study had 3 arms, including 2 intervention and 1 control.^24^ The other 17 included studies had 2 arms (intervention and control).

Six studies were conducted in South Africa,^22,23,29,48,54,55^ 1 in both South Africa and Malawi,^28^ 4 in Nigeria,^17,20,49,53^ 2 in Kenya,^21,24^ 1 in Ghana,^27^ 1 in Mali,^18^ 1 in Ethiopia,^50,51^ 1 in Rwanda,^52^ and 1 in Senegal.^56^ Most studies recruited participants from hospital clinic settings,^17,20,24,27,48–55^ some from community health center settings,^18,21–23,29,56^ and 1 from both hospital clinic and community health center settings.^28^ Delineating urban vs rural settings was not feasible given the wide geographical areas that many of the involved health care facilities served. Sample sizes ranged from 46 to 1570. One study recruited only women,^54^ and in the remaining 17 studies, 15.5% to 67.2% of participants were male. Where mean (SD) age of study participants was available, this ranged from 48.8 (9.8) to 58.8 years (7.7).^21,23^ Where median (IQR) age was available, this ranged from 53 (41–58) to 60 years (54–69) in the intervention groups and 50 (39–62) to 62 years (56–69) in the control groups.^29,52^ Participants in the included studies had pre-existing T2DM and were on a range of treatments, including diet control only, oral antidiabetic drugs, and insulin. Eight studies reported on diabetic complications and comorbidities among participants, which included hypertension, dyslipidemia, ischemic heart disease, heart failure, transient ischemic attack and stroke, peripheral vascular disease, chronic kidney disease, tuberculosis, mental illness, cataracts, retinopathy, leg ulcers, neuropathy, foot disease, amputation, asthma, epilepsy, HIV/AIDS, arthritis, and gastrointestinal illness.

Eight of the studies assessed T2DM self-management education programs covering a broad range of self-management topics,^17,21,22,27,49–51,53,55^ 2 assessed peer educator/support interventions,^18,29^ 2 assessed exercise interventions with additional exercise-related education components or problem-solving,^20,54^ 2 assessed nutrition education interventions,^23,48^ 2 assessed automated educational text messaging–based interventions,^28,56^ 1 assessed a blood glucose self-monitoring intervention,^52^ and 1 assessed a nutrition education program in 1 intervention arm with the same nutrition education program plus peer support in the second intervention arm.^24^

The self-management interventions in the included studies were found to span multiple categories of the PRISMS taxonomy, rather than each having a single primary category. For each study, 1 to 3 categories of the PRISMS taxonomy applied (Appendix IV). The categories with the greatest coverage were category xiv (lifestyle advice and support; 13 studies)^18,20–24,27,29,48–51,54,55^ and category i (education about condition and management; 12 studies).^17,18,21–24,27,29,48–51,53^ These 2 were followed by category vi (practical support with medication or behavioral adherence; 3 studies),^17,28,53^ category xiii (social support; 3 studies),^18,24,29^ category xi (training/rehearsal for practical self-management activities; 2 studies),^52,54^ category v (monitoring of condition with feedback to the patient; 1 study),^52^ and category xii (training/rehearsal for psychological strategies; 1 study).^20^

Duration of intervention ranged from a one-off training session to education spread across 1 year, with variable frequency of sessions. Self-management interventions were delivered face-to-face in 11 studies,^17,18,20,22–24,29,48–51,53^ remotely in 3 studies,^27,28,56^ and both face-to-face and remotely in 4 studies.^21,52,54,55^ Interventions were individual (one-to-one) in 5 studies,^21,27,28,49,56^ group-based in 6 studies,^18,22–24,50,51,55^ a combination of individual and group-based in 3 studies,^29,48,54^ and unclear in 4 studies.^17,20,52,53^ Interventions were delivered by health care professionals in 9 studies, including 3 delivered by pharmacists,^17,49,53^ 2 by dieticians,^23,48^ 2 by nurses,^27,50,51^ 1 by certified diabetes educators,^21^ and 1 by unspecified mid-level health workers.^22^ In 1 study, the intervention was delivered by both health care professional and lay-persons^24^; in 2 studies, it was delivered by lay-persons^18,29^; in 4 studies, it was unclear who was delivering the intervention^20,52,54,55^; and in 2 studies, it was based on automated text messaging and therefore not delivered by a specified person.^28,56^ Regarding comparator, the control group in 11 studies consisted of usual care (with no change),^17,18,21,22,29,49–53,55,56^ a form of enhanced usual care (changes or additions to usual care) in 5 studies,^20,23,24,27,48^ and a sham intervention (designed to act as a placebo) in 2 studies, which consisted of a sham text-messaging intervention^28^ and a relaxation intervention.^54^

When conducting the review, it became apparent that there were no 24-month outcome data available. However, several studies included 3-month and 6-month outcome data. On that basis, an additional 3-month time point for data extraction was included, which was a deviation from the protocol.

### Review findings

#### Primary outcomes

##### HbA1c

**Figure 2:**
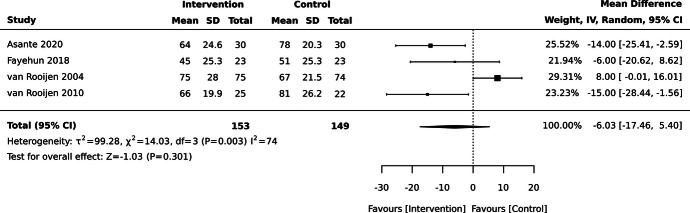
Effect of self-management interventions on glycated hemoglobin (HbA1c; mmol/mol) at 3 months in adults with type 2 diabetes mellitus in sub-Saharan Africa. Asante 2020^27^: Individually randomized, parallel-design randomized controlled trial (RCT) set in Ghana, assessing a broad self-management education program compared with enhanced usual care. Fayehun 2018^20^: Individually randomized, parallel-design RCT set in Nigeria, assessing an exercise intervention with counseling and problem-solving compared with enhanced usual care. van Rooijen 2004^54^: Individually randomized, parallel-design RCT set in South Africa, assessing an exercise intervention with education compared with a relaxation-based sham intervention. van Rooijen 2010^55^: Individually randomized, parallel-design RCT set in South Africa, assessing a broad self-management education program compared with usual care.

Self-management interventions did not significantly reduce average HbA1c levels at 3 months compared with the control, based on meta-analysis of 302 participants from 4 studies (MD –6.0 mmol/mol, 95% CI –17.5, 5.4; see Figure 2).^20,27,54,55^ Additionally, a cluster RCT assessing the effectiveness of a peer-support self-management program compared with usual care did not show a significant reduction in average HbA1c at 4 months, in keeping with the findings of the meta-analysis.^29^ A cluster randomized crossover trial assessing the effectiveness of an educational text messaging intervention did suggest a reduction in average HbA1c in the intervention group vs control (usual care) pre-crossover at 3 months, although with some heterogeneity in intervention type vs the other included studies.^56^ Of note, there was significant heterogeneity in the meta-analysis (*I* ^2^ = 74%) with 1 discordant study trending toward favoring control.^54^

**Figure 3:**
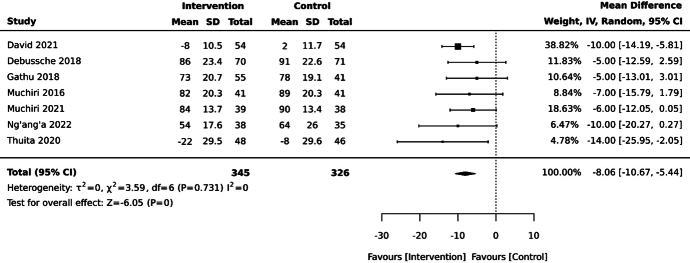
Effect of self-management interventions on glycated hemoglobin (HbA1c; mmol/mol) at 6 months in adults with type 2 diabetes mellitus in sub-Saharan Africa. David 2021^49^: Individually randomized, parallel-design randomized controlled trial (RCT) set in Nigeria, assessing a broad self-management education program compared with usual care. Debussche 2018^18^: Individually randomized, parallel-design RCT set in Mali, assessing a peer-support intervention compared with usual care. Gathu 2018^21^: Individually randomized, parallel-design RCT set in Kenya, assessing a broad self-management education program compared with usual care. Muchiri 2016^23^: Individually randomized, parallel-design RCT set in South Africa, assessing a nutrition education intervention compared with enhanced usual care. Muchiri 2021^48^: Individually randomized, parallel-design RCT set in South Africa, assessing a nutrition education intervention compared with enhanced usual care. Ng’ang’a 2022^52^: Individually randomized, parallel-design RCT set in Rwanda, assessing a blood glucose self-monitoring intervention compared with usual care. Thuita 2020^24^: Individually randomized, parallel-design RCT with 3 arms set in Kenya; assessing nutrition education program plus peer support compared with enhanced usual care included in this meta-analysis.

Self-management interventions reduced average HbA1c levels by 8.1 mmol/mol (95% CI –10.7, –5.4) at 6 months compared with the control, based on meta-analysis of 671 participants from 7 studies (Figure 3).^18,21,23,24,48,49,52^ One of these studies had 2 intervention arms, including a nutrition education program, and the same program with additional peer support.^24^ The more comprehensive nutrition education plus peer support intervention has been included in the main meta-analysis models, with a sensitivity analysis conducted using the nutrition education only arm.

**Figure 4:**
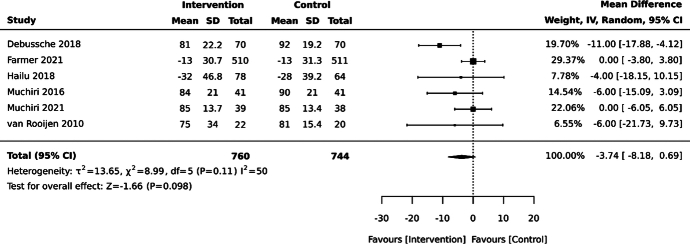
Effect of self-management interventions on glycated hemoglobin (HbA1c; mmol/mol) at 12 months in adults with type 2 diabetes mellitus in sub-Saharan Africa. Debussche 2018^18^: Individually randomized, parallel-design randomized controlled trial (RCT) set in Mali, assessing a peer-support intervention compared with usual care. Farmer 2021^28^: Individually randomized, parallel-design RCT set in South Africa and Malawi, assessing an automated educational text messaging–based intervention compared with a text messaging sham intervention. Hailu 2018^50^: Individually randomized, parallel-design RCT set in Ethiopia, assessing a broad self-management education program compared with usual care. Muchiri 2016^23^: Individually randomized, parallel-design RCT set in South Africa, assessing a nutrition education intervention compared with enhanced usual care. Muchiri 2021^48^: Individually randomized, parallel-design RCT set in South Africa, assessing a nutrition education intervention compared with enhanced usual care. van Rooijen 2010^55^: Individually randomized, parallel-design RCT set in South Africa, assessing a broad self-management education program compared with usual care.

Self-management interventions did not significantly reduce average HbA1c levels at 12 months compared with the control, based on meta-analysis of 1504 participants from 6 studies (MD –3.7 mmol/mol, 95% CI –8.2, 0.7; see Figure 4).^18,23,28,48,50,55^ A further cluster RCT assessing a group-based diabetes education program did not show a significant difference in post-intervention average HbA1c compared with usual care; however, successful delivery of the intervention was low, with 59.4% of participants in the intervention group not attending any of the education sessions.^22^ The *I* ^2^ statistic showed heterogeneity of 50% in this meta-analysis.

##### Fasting blood glucose

**Figure 5:**
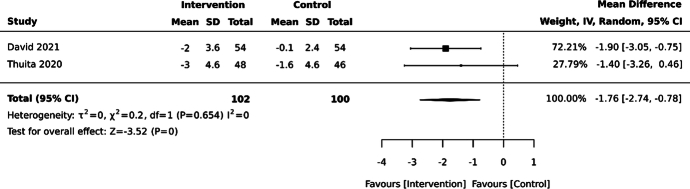
Effect of self-management interventions on fasting blood glucose (mmol/L) at 6 months in adults with type 2 diabetes mellitus in sub-Saharan Africa. David 2021^49^: Individually randomized, parallel-design randomized controlled trial (RCT) set in Nigeria, assessing a broad self-management education program compared with usual care. Thuita 2020^24^: Individually randomized, parallel-design RCT with 3 arms set in Kenya; nutrition education program plus peer support compared with enhanced usual care included in this meta-analysis.

Three studies assessed the effect of self-management interventions on FBG in 353 participants. Based on pre-set outcome time point combinations, 2 studies (202 participants) were combined in meta-analysis showing a reduction in average FBG levels at 6 months (MD –1.8 mmol/L, 95% CI –2.7, –0.8; see Figure 5).^24,49^ The results of the third study were similar.^17^

##### Health-related quality of life

Four studies (2205 participants) measured the effect of self-management interventions on HRQoL, and their combined findings are presented in Table 2.^22,28,51,53^ These were synthesized narratively, as a combination of individually vs cluster randomized study designs, change from baseline vs post-intervention data, and outcomes reported at different time points, precluded meta-analysis of standardized MD for this outcome measure. Three studies found no significant effect of self-management interventions on average HRQoL compared with the control,^22,28,50^ while 1 did find a significant improvement for all HRQoL domains in the RAND-36 Item Health Survey, apart from pain.^53^ The discordant study had methodological constraints, scoring “yes” for only 1 of 13 criteria in the standardized JBI critical appraisal tool for RCTs.^53^ The synthesized findings therefore do not appear to support a significant effect of self-management interventions on HRQoL outcomes among people living with T2DM in sub-Saharan Africa from the limited available data.

**Table 2:** Effect of self-management interventions on health-related quality of life among adults with type 2 diabetes mellitus in sub-Saharan Africa

Study	Farmer et al. 2021^28^	Hailu et al. 2021^51^	Mash et al. 2014^22^	Ojieabu 2020^53^
**Design**	Standard RCT	Standard RCT	Cluster RCT	Standard RCT
**Questionnaire**	EQ-5D-3L	HowRU tool	SF-20	RAND-36 Item Health Survey
**Direction**	Higher score = higher HRQoL	Higher score = higher HRQoL	Higher score = higher HRQoL	Higher score = higher HRQoL
**Time point**	12 months	9 months	12 months	4 months 8 months
**Data type**	Change from baseline	Change from baseline	Post intervention	Post intervention
**Intervention group (n)**	513	78	391	85
**Control group (n)**	514	64	475	85
**Intervention group mean (SD)**	0.009 (0.160)	0.6 (2.76)	Physical functioning 26.4 (6.1) Role functioning 81.7 (25.6) Social functioning 63.2 (30.8) Mental health 60.1 (13.7) General health 58.8 (10.8) Pain 57.7 (29.9)	Each domain has 2 x mean (SD). The first value is for participants who were taking 1-5 pills, and the second for those taking >5 pills (of any type).* 4-month data: Physical functioning 67.6 (23.1); 63.6 (18.8) Role limitations due to physical health 54.2 (12.8); 46.7 (39.7) Role limitations due to emotional problems 53.4 (13.6); 48.3 (46.2) Energy/fatigue 50.4 (10.0); 50.9 (11.7) Emotional well-being 48.2 (12.4); 42.7 (11.5) Social functioning 52.1 (11.7); 60.8 (21.6) Pain 53.9 (22.1); 66.2 (26.9) General health 66.6 (18.6); 61.8 (21.3) 8-month data: Physical functioning 92.5 (3.5); 75.7 (14.2) Role limitations due to physical health 91.5 (28.2); 83.5 (23.5) Role limitations due to emotional problems 90.0 (21.4); 87.8 (33.1) Energy/fatigue 60.0 (14.1); 55.4 (8.0) Emotional well-being 60.0 (14.1); 62.8 (7.6) Social functioning 81.3 (26.5); 65.2 (16.4) Pain 55.7 (23.8); 62.4 (20.9) General health 77.5 (10.6); 67.8 (12.8)
**Control group mean (SD)**	–0.004 (0.157)	1.14 (3.23)	Physical functioning 26.9 (6.0) Role functioning 79.1 (26.4) Social functioning 63.7 (30.4) Mental health 60.2 (13.2) General health 60.0 (11.4) Pain 56.0 (30.5)	Each domain has 2 x mean (SD). The first value is for participants who were taking 1-5 pills, the second for those taking >5 pills. 4-month data: Physical functioning 47.2 (19.1); 52.2 (9.6) Role limitations due to physical health 24.2 (11.4); 36.9 (31.7) Role limitations due to emotional problem 23.4 (16.2); 35.0 (34.2) Energy/fatigue 36.4 (16.5); 38.1 (12.2) Emotional well-being 29.2 (14.2); 37.7 (13.3) Social functioning 42.1 (17.5); 50.1 (11.6) Pain 47.3 (18.2); 42.8 (23.3) General health 50.2 (16.1); 58.3 (19.2) 8-month data: Physical functioning 47.7 (16.1); 56.5 (11.1) Role limitations due to physical health 31.3 (26.3); 35.1 (21.2) Role limitations due to emotional problems 28.0 (23.3); 37.3 (23.8) Energy/fatigue 37.3 (24.1); 37.4 (7.8) Emotional well-being 32.0 (11.1); 38.4 (8.4) Social functioning 39.3 (22.7); 50.1 (11.6) Pain 51.8 (18.1); 41.4 (18.9) General health 54.0 (13.3); 60.8 (9.6)
**Comment**	Authors found no statistically significant difference between intervention and control group HRQoL scores.	Authors found no statistically significant difference between intervention and control group HRQoL scores.	Authors found no statistically significant difference between intervention and control group HRQoL scores.	Authors found a statistically significant improvement in HRQoL scores with the intervention in all domains, apart from pain.

* Ojieabu 2020^53^ split the analysis by pill burden, separating those taking 1–5 pills from those taking >5 pills.

EQ-5D-3L, European Quality of Life 5 Dimensions 3 Level Version; HRQoL, health-related quality of life; RCT, randomized controlled trial; SF-20, 20-Item Short Form Health Survey.

##### Adverse effects

Only 3 studies specifically reported on adverse events among 1217 participants combined, and none related to the trial interventions were reported.^28,54,55^ As no studies reported any adverse effects relating to the trial interventions, it was not possible to present risk ratios with 95% CIs for this outcome measure. One study assessing an automated educational text messaging intervention compared to a sham text intervention reported 19 (3.4%) deaths in the intervention group and 16 (2.9%) deaths in the control group, with no adverse events related to the trial intervention at 12 months.^28^ Studies assessing a home-based exercise intervention with peer-support and exercise-related education^54^ and a group-delivered diabetes education program^55^ both reported no adverse events in intervention and control groups over 12 weeks and 1 year of follow-up, respectively.

#### Secondary outcomes

Individual forest plots for the secondary outcomes of weight, BMI, waist circumference, SBP, DBP, and lipids are available in Appendix V.

##### Weight

Meta-analysis assessing the effect of self-management interventions on weight at 6 months in 2 studies (235 participants) found that average weight was reduced by 6.1 kg compared with the control (95% CI –8.8, –3.4; see Table 3).^18,24^ One of these studies also followed up at 12 months, where this effect persisted.^18^ A cluster RCT that was not able to be combined in meta-analysis did not demonstrate a significant effect on average weight at 12 months; however, successful delivery of the intervention was low with 59.4% of participants in the intervention group not attending any of the education sessions.^22^

**Table 3:** Summary of meta-analysis results for weight, body mass index, systolic blood pressure, and diastolic blood pressure

Outcome measure	Outcome time point	Meta-analysis: results Mean difference (95% CI) Statistical heterogeneity (*I* ^2^ statistic, %)	Meta-analysis: total participants and included studies
Weight (kg)	6 months	–6.08 (–8.76, –3.40) *I* ^2^ = 21	235 participants: Debussche,^18^ 2018 Thuita,^24^ 2020
Body mass index (kg/m^2^)	3 months	–0.66 (–2.10, 0.77) *I* ^2^ = 0	345 participants: Oijeabu,^17^ 2017 van Rooijen,^54^ 2004 van Rooijen,^55^ 2010
	6 months	–0.98 (–1.97, 0.01) *I* ^2^ = 83	490 participants: Debussche,^18^ 2018 Gathu,^21^ 2018 Muchiri,^23^ 2016 Muchiri,^48^ 2021 Thuita,^24^ 2020
	12 months	–0.33 (–1.03, 0.37) *I* ^2^ = 61	1343 participants: Debussche,^18^ 2018 Farmer,^28^ 2021 Muchiri,^23^ 2016 Muchiri,^48^ 2021 Van Rooijen,^55^ 2010
Systolic blood pressure (mmHg)	3 months	–6.56 (–16.36, 3.24) *I* ^2^ = 84	299 participants: Oijeabu,^17^ 2017 van Rooijen,^54^ 2004
	6 months	–2.35 (–5.13, 0.44) *I* ^2^ = 10	598 participants: David,^49^ 2021 Debussche,^18^ 2018 Gathu,^21^ 2018 Muchiri,^23^ 2016 Muchiri,^48^ 2021 Thuita,^24^ 2020
	12 months	–3.80 (–6.19, –1.40) *I* ^2^ = 12	1317 participants: Debussche,^18^ 2018 Farmer,^28^ 2021 Muchiri,^23^ 2016 Muchiri,^48^ 2021
Diastolic blood pressure (mmHg)	3 months	–3.96 (–10.72, 2.80) *I* ^2^ = 89	299 participants: Oijeabu,^17^ 2017 van Rooijen,^54^ 2004
	6 months	–1.00 (–3.77, 1.77) *I* ^2^ = 67	598 participants: David,^49^ 2021 Debussche,^18^ 2018 Gathu,^21^ 2018 Muchiri,^23^ 2016 Muchiri,^48^ 2021 Thuita,^24^ 2020
	12 months	–1.41 (–3.15, 0.33) *I* ^2^ = 43	1317 participants: Debussche,^18^ 2018 Farmer,^28^ 2021 Muchiri,^23^ 2016 Muchiri,^48^ 2021

##### Body mass index

No significant effect of self-management interventions on average BMI was found at 3, 6, or 12 months (Table 3), or in an additional cluster RCT at the 3-month time point.^29^ There was substantial heterogeneity in the meta-analyses for the 6- and 12-month time points (*I* ^2^ = 83% and 61%, respectively).

##### Waist circumference

For the outcome of WC, meta-analysis was not possible due to a combination of individually and cluster randomized RCTs. At 6 months, 1 study assessing a nutrition education intervention plus peer support found a marked reduction in average WC compared with the control (MD –16.5 cm, 95% CI –20.2, –12.7),^24^ whereas a cluster RCT assessing the effect of a peer-support intervention did not find a significant difference in average WC between intervention and control groups.^29^ The effect at 12 months was also contradictory. One study demonstrated mean WC decreased by 3.3 cm (95% CI –5.6, –1.1) in the intervention group and increased by 2.7 cm (95% CI 0.2, 5.1) in the control group,^18^ while another study found no significant difference in WC between intervention and control groups, albeit with a low rate of intervention delivery.^22^

##### Systolic blood pressure

When assessed by meta-analysis, the effect of self-management interventions on average SBP was not significant compared with the control at 3 or 6 months (Table 3). There was substantial heterogeneity in the 3-month meta-analysis (*I* ^2^ = 84%). Data from an additional cluster RCT at the 3-month time point also showed no effect on SBP.^29^ A statistically significant but small effect on average SBP was seen at 12 months in meta-analysis (1317 participants, MD –3.8 mmHg, 95% CI –6.2, –1.4; see Table 3) and in a further cluster RCT.^22^ This was of unlikely clinical significance, accepting a minimum clinically significant difference of 5 mmHg.^57^

##### Diastolic blood pressure

When assessed by meta-analysis, the effect of self-management interventions on average DBP was not significant compared with the control at 3, 6, or 12 months (Table 3). Heterogeneity was substantial in the 3- and 6-month meta-analysis (*I* ^2^ = 89% and 67%, respectively). Small improvements in DBP of unlikely clinical significance (accepting a minimum clinically significant difference of 5 mmHg)^57^ were seen in a cluster RCT at 3 months^29^ and a separate cluster RCT at 12 months,^22^ which could not be included in meta-analysis.

##### Lipids

No significant effect of self-management interventions on lipid profile was found, except for a small reduction in LDL at 6 months in a meta-analysis of 4 studies (361 participants, MD –0.30 mmol/L, 95% CI –0.57, –0.02). Given the upper end of the 95% CI approaches zero and the size of the effect estimate is small, this was of unlikely clinical significance. Results for synthesis of lipid profile are presented in Table 4.

**Table 4:** Effect of self-management interventions on lipids among adults with type 2 diabetes mellitus in sub-Saharan Africa

Lipid measure	Outcome time point	Meta-analysis: results Mean difference (95% CI) Statistical heterogeneity (*I* ^2^ statistic, %)	Meta-analysis: total participants and included studies	Data from additional studies not suitable for meta-analysis: Mean (SD), total participants
Total cholesterol (mmol/L)	3 months	N/A	N/A	van Rooijen,^55^ 2010: I = 4.67 (1.06), 25 C = 4.69 (1.26), 22
	6 months	–0.25 (–0.66, 0.16) *I* ^2^ = 82	361 participants: David,^49^ 2021 Muchiri,^23^ 2016 Muchiri,^48^ 2021 Thuita 2020	N/A
	12 months	–0.22 (–0.44, 0.00) *I* ^2^ = 0	202 participants: Muchiri,^23^ 2016 Muchiri,^48^ 2021 Van Rooijen,^55^ 2010	Mash,^22^ 2014*: I = 4.80 (1.10), 391 C = 4.90 (1.20), 475
High-density lipoprotein cholesterol (mmol/L)	3 months	N/A	N/A	van Rooijen,^55^ 2010: I = 0.98 (0.25), 25 C = 1.06 (0.32), 22
	6 months	0.00 (–0.07, 0.08) *I* ^2^ = 44	361 participants: David,^49^ 2021 Muchiri,^23^ 2016 Muchiri,^48^ 2021 Thuita,^24^ 2020	N/A
	12 months	–0.09 (–0.17, 0.00) *I* ^2^ = 29	202 participants: Muchiri,^23^ 2016 Muchiri,^48^ 2021 Van Rooijen,^55^ 2010	N/A
Low-density lipoprotein cholesterol (mmol/L)	3 months	N/A	N/A	van Rooijen,^55^ 2010: I = 2.88 (0.96), 25 C = 2.6 (1.15), 22
	6 months	–0.30 (–0.57, –0.02) *I* ^2^ = 67	361 participants: David,^49^ 2021 Muchiri,^23^ 2016 Muchiri,^48^ 2021 Thuita,^24^ 2020	N/A
	12 months	–0.22 (–0.56, 0.12) *I* ^2^ = 54	202 participants: Muchiri,^23^ 2016 Muchiri,^48^ 2021 Van Rooijen,^55^ 2010	N/A
Triglycerides (mmol/L)	3 months	N/A	N/A	van Rooijen,^55^ 2010: I = 1.80 (1.05), 25 C = 1.82 (2.21), 22
	6 months	0.19 (–0.04, 0.42) *I* ^2^ = 36	279 participants: David,^49^ 2021 Muchiri,^48^ 2021 Thuita,^24^ 2020	N/A
	12 months	0.18 (–0.36, 0.73) *I* ^2^ = 43	120 participants: Muchiri,^48^ 2021 Van Rooijen,^55^ 2010	N/A

C, control group; I, intervention group; N/A, not applicable.

*Not included in meta-analysis due to cluster design and no intracluster correlation coefficient.

#### Subgroup and sensitivity analyses

Two meta-analyses showed significant improvement in a primary outcome and were therefore potentially eligible for subgroup analysis: HbA1c at 6 months and FBG at 6 months. However, for the FBG at 6 months meta-analysis, there were only 2 included studies, precluding subgroup analysis. This left only the HbA1c at 6 months meta-analysis for subgroup analysis (Appendix VI).

The first subgroup analysis compared the effect of usual care vs enhanced usual care as the comparator (none of the studies included in the HbA1c at 6 months meta-analysis had a sham intervention as their comparator). The second subgroup analysis compared group-based self-management interventions vs those delivered individually. In both cases, there was no indication of significant subgroup differences (*P*=0.76 and 0.70, respectively).

Subgroup analysis based on the primary category of self-management intervention according to the PRISMS taxonomy was not feasible due to interventions spanning multiple categories.^9^ There were insufficient data to investigate face-to-face vs remote delivery method, or intervention delivery by professional vs lay person.

Reduction in HbA1c at 6 months with self-management intervention vs control remained significant when studies of poor methodological quality were removed from the meta-analysis, as well as for those that were commercially funded (Appendix VI). Sensitivity analyses based on excluding studies that were not written in English or not a journal publication (ie, not peer-reviewed) were not applicable.

When the nutrition education intervention arm from the 3-arm study^24^ was included in meta-analysis models in lieu of the nutrition education plus peer support intervention arm, similar findings were produced for the outcomes of HbA1c, FBG, weight, SBP, DBP, total cholesterol, HDL, and LDL at 6 months. The MD for BMI at 6 months became marginally significant at –0.4 kg/m^2^ (491 participants, 95% CI –0.8, –0.1). The MD for triglycerides at 6 months favored control (280 participants, 0.29 mmol/L, 95% CI 0.17, 0.41). Full results for this sensitivity analysis are presented in Appendix VI.

As there were not 10 or more studies included in a single meta-analysis, a funnel plot was not generated to assess for publication bias.

## Discussion

The findings of this systematic review suggest that although self-management interventions for people living with T2DM in sub-Saharan African settings may produce a significant reduction in HbA1c at 6 months, there was no significant effect on HbA1c at 12 months and no 24-month outcome data available. For the 6-month effect estimate, there was low confidence on the GRADE assessment due to a lack of blinding and risk of attrition bias, while there was moderate confidence in the 12-month estimate. The 95% CIs for the effect on HbA1c at 12 months ranged from a lower limit of an 8.2 mmol/mol reduction to an upper limit of an 0.7 mmol/mol increase, and it is possible that the interventions are still somewhat effective at the 12-month time point. Nevertheless, even if an effect does persist, the magnitude of that effect appears reduced by 12 months compared with 6 months. The findings are consistent with existing umbrella review evidence from a variety of geographical settings outside of sub-Saharan Africa, where a reduction in HbA1c with self-management interventions was reported at 6 months but appeared attenuated by 12 months.^11^ With complex interventions such as those targeting self-management behaviors, there is a risk that blanket application to alternative contexts without appropriate tailoring of the intervention may cause the intervention to fail. The consistency of our findings with those from other settings may be suggestive of the interventions having been adapted appropriately, with reproducible beneficial effects on HbA1c in different groups and contexts.^11–14^

In addition, no significant effect on HbA1c was found at 3 months from intervention onset, although with very low confidence in this finding due to lack of blinding, small sample sizes, and statistical heterogeneity. HbA1c is a measure of longer-term diabetic control,^1^ and there may have been inadequate time for the intervention to be reflected in significant HbA1c changes at 3 months. Additionally, there was substantial heterogeneity in the meta-analysis (*I* ^2^ = 74%) with 1 discordant study trending toward favoring control.^54^ This may have been due to the presence of a sham intervention (relaxation program) in the discordant study, which could feasibly confer some benefit to overall well-being and, therefore, T2DM management. FBG as an alternative measure of glycemic control also showed a small reduction at 6 months with self-management interventions vs control, although without 12- or 24-month outcome data and with very low confidence on the GRADE assessment. It is possible that the effect of self-management interventions for T2DM on glycemic control wanes over time, for example, through reduced intervention adherence.

This systematic review found that self-management interventions did not appear to improve HRQoL for people with T2DM in sub-Saharan Africa at 4 and 12 months; however, data were limited and unsuitable for meta-analysis, and there was low certainty on the GRADE assessment due to lack of blinding and inconsistency in results among the included studies.^22,28,50,53^ Evidence from other settings is generally suggestive of a benefit.^58–60^ Several factors may have influenced the conflicting findings. In a trial of a nurse-led education program that showed no improvement in HRQoL, the authors note that low literacy among participants and the nonspecific nature of the chosen HRQoL measurement tool may have biased the findings in favor of no benefit.^50^ In a further study of a group-based education program that found no benefit on HRQoL, 59.4% of participants did not attend a single education session, markedly increasing the risk of type II error.^22^ Conversely, the study that did demonstrate a beneficial effect of self-management interventions on HRQoL had methodological constraints, scoring a “yes” for only 1 out of 13 questions on the JBI standardized critical appraisal tool for RCTs.^53^ Most studies included in this review (14/18) did not report on any HRQoL measure. This and other patient-reported outcome measures should be prioritized in future studies of self-management interventions in this context.

Only 3 out of 18 trials reported on adverse events, stating that none occurred in relation to the trial interventions. There were insufficient data overall to confidently determine the effect of self-management interventions on adverse events. The reporting of adverse events in trials of self-management interventions may not be as crucial as in, for example, trials of pharmacological interventions; however, they should still be reported to provide a balanced picture. Examples of adverse events that could feasibly occur in self-management interventions include injury from exercise or hypoglycemia resulting from dietary and exercise advice.

There is uncertainty in the findings regarding the primary outcome measures ranging from very low to moderate confidence on the GRADE assessment. This was partially driven by limitations of the included studies resulting in increased risk of bias. While this could reflect true methodological limitations among the included studies, reporting limitations could also account for some of the lower scores on the critical appraisal. Further, blinding of both participants and those delivering interventions is challenging for complex self-management interventions and was not widely applied, risking bias in favor of finding an effect.

Several secondary outcomes measures were assessed in this review (weight, BMI, WC, SBP, DBP, total cholesterol, HDL, LDL, and triglycerides). Statistical heterogeneity was substantial in several of the meta-analyses of secondary outcomes. There was a significant reduction in weight at 6 months on meta-analysis of 2 studies, which was not reflected in the meta-analyses of effect on BMI (no significant reduction at 3, 6, or 12 months). Further, data on the effect of self-management interventions on WC were contradictory and not suitable for meta-analysis. The mixed picture across these 3 linked physiological outcome measures may be explained by heterogeneity among the studies reporting on different outcome measures at different time points, such as differences in study interventions. The broad definition of self-management interventions applied in this systematic review^9^ introduces some inevitable heterogeneity among the type of interventions being assessed, but must be balanced against the need to avoid overly restrictive inclusion criteria.^61^ There is some inherent tension between the need for self-management interventions to be tailored to their unique context and proposed recipients vs the need for collective evidence of their effectiveness to inform decision-making and policy regarding their application.

Among the remaining secondary outcome measures of blood pressure and lipids, no significant effect of self-management interventions was demonstrated, apart from small reductions in SBP at 12 months and LDL at 6 months (MD –3.8mmHg, 95% CI –6.2, –1.4 and MD –0.3 mmol/L, 95% CI –0.6, –0.02, respectively). These were felt to be of unlikely clinical significance.^57,62^

Although subgroup analysis indicated there were no significant differences in subgroups based on comparator for the meta-analysis of HbA1c at 6 months (Appendix VI), it is still important to acknowledge the heterogeneity among comparators in the included studies. The comparator was usual care for the majority of studies, although in some studies there were changes or additions to usual care (termed “enhanced usual care”),^20,23,24,27,48^ and 2 studies made use of a sham intervention, namely a sham text-messaging intervention^28^ and a relaxation intervention.^54^ The variation in comparator could still impact the results. It is possible for a sham intervention to confer some inherent benefit itself, for example, by improving overall well-being and, therefore, diabetes management. This may reduce the ability of a study to detect a true effect of the intervention. There may also be differences of opinion on what constitutes a sham intervention; however, where this term was used, a description of what was involved was provided, and this is unlikely to have significantly affected interpretation of the results.

In addition, where means and SDs were not reported in the included studies, we estimated these based on the median, standard error, 95% CIs, and IQRs. Information on the distribution of the data was often not available in the included studies. There is a risk that inaccuracies in the estimated means and SDs could affect the relative weighting of studies in the meta-analyses.

A further limitation of our review was that the available evidence meeting our inclusion criteria came from only 9 of the 48 eligible sub-Saharan African countries. This lack of breadth constrains generalizability across the wide and varied sub-Saharan African context. Heterogeneity among the included populations in terms of geographical and sociocultural context also constrains generalizability.

To the reviewers’ knowledge, this systematic review provides the most up-to-date evidence synthesis of the effectiveness and safety of self-management interventions among people living with T2DM in sub-Saharan Africa. Comprehensive search strategies across a wide range of databases have ensured that the breadth of available RCTs on this topic were accessed. A strength of the review is the inclusion of patient-centered outcome measures such as HRQoL and adverse events. However, this has highlighted a paucity of data for these outcome measures, and the need for increased reporting of such outcomes in future trials of self-management interventions in the sub-Saharan African context.

## Conclusions

Self-management interventions for adults living with T2DM in sub-Saharan Africa may produce a clinically significant improvement in glycemic control at 6 months (low certainty on GRADE assessment), but this may wane in the longer-term (moderate certainty on GRADE assessment). There was not convincing evidence of a benefit of these interventions on HRQoL (low certainty on GRADE assessment), but reporting on this outcome measure was limited. There were insufficient data on adverse events to draw conclusions (low certainty on GRADE assessment). Recommendations are rated according to the JBI Grades of Recommendation.^63^

### Recommendations for practice and policy

The provision of self-management interventions for people living with T2DM in the sub-Saharan African context should be considered as part of an overall management strategy (Grade B); however, consideration should be given to the longevity of interventions and methods to maintain adherence over time, given the potential for waning of effects.

### Recommendations for research

Given the limitations of the available evidence and to strengthen the evidence base, high-quality RCTs should be conducted and reported, with a particular focus on longer-term follow-up and on patient-reported outcomes such as HRQoL. Given the potential for the effects of self-management interventions to wane over time, qualitative exploration of barriers and facilitators of long-term adherence to self-management interventions should be considered.

## Acknowledgments

Alison Ashmore, senior research librarian at the University of Nottingham (UK), for her contribution to the search strategies.

## Funding

NC was an In-Practice Fellow supported by the UK’s Department of Health and Social Care and the National Institute for Health Research (NIHR301000). The views expressed are those of the authors and not necessarily those of the NHS, the NIHR, or the Department of Health and Social Care.

## Author contributions

Planning and design of the review was undertaken by NC, PC, and KC. Data extraction and synthesis was undertaken by NC and GN, and where necessary, discussed with KC. Initial manuscript drafting was undertaken by NC, and all authors contributed to manuscript review and editing. Senior oversight of the project was provided by KC.

## Data availability statement

The data extracted from studies are available from the corresponding author.

## Figures and Tables

**Figure S1:**
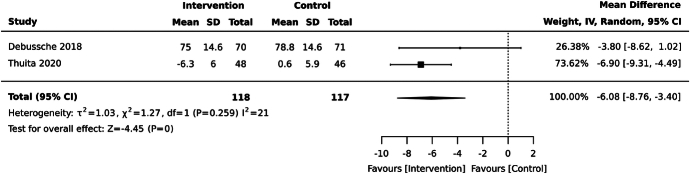
Effect of self-management interventions on weight (kg) at 6 months in adults with type 2 diabetes mellitus in sub-Saharan Africa

**Figure S2:**
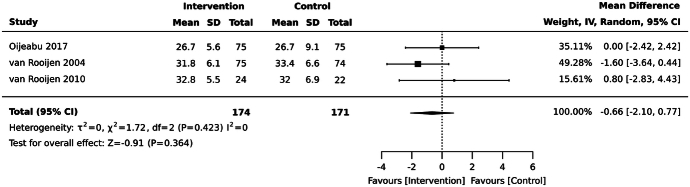
Effect of self-management interventions on body mass index (kg/m^2^) at 3 months in adults with type 2 diabetes mellitus in sub-Saharan Africa

**Figure S3:**
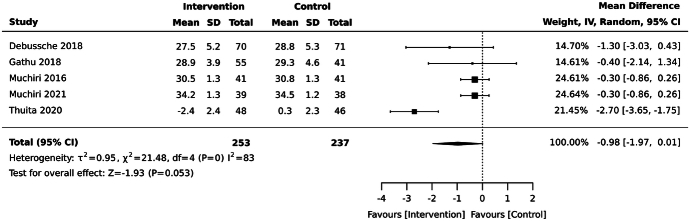
Effect of self-management interventions on body mass index (kg/m^2^) at 6 months in adults with type 2 diabetes mellitus in sub-Saharan Africa

**Figure S4:**
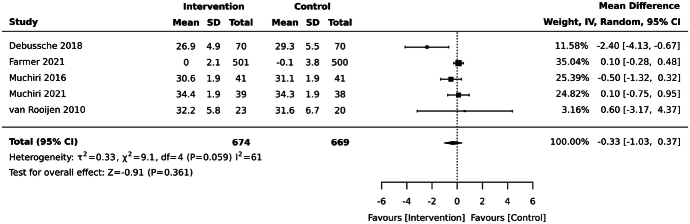
Effect of self-management interventions on body mass index (kg/m^2^) at 12 months in adults with type 2 diabetes mellitus in sub-Saharan Africa

**Figure S5:**
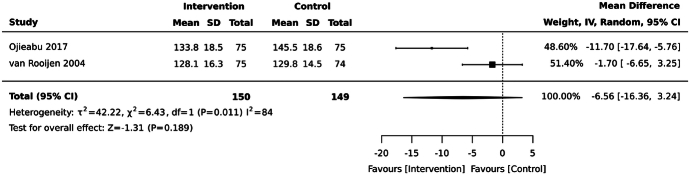
Effect of self-management interventions on systolic blood pressure (mmHg) at 3 months in adults with type 2 diabetes mellitus in sub-Saharan Africa

**Figure S6:**
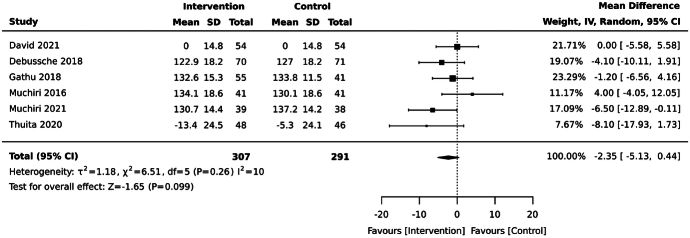
Effect of self-management interventions on systolic blood pressure (mmHg) at 6 months in adults with type 2 diabetes mellitus in sub-Saharan Africa

**Figure S7:**
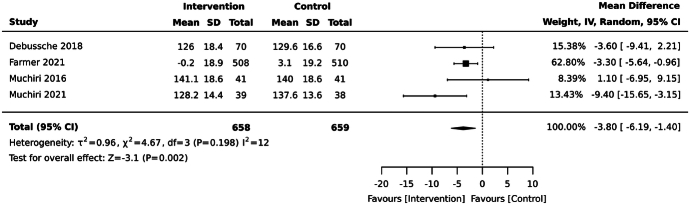
Effect of self-management interventions on systolic blood pressure (mmHg) at 12 months in adults with type 2 diabetes mellitus in sub-Saharan Africa

**Figure S8:**
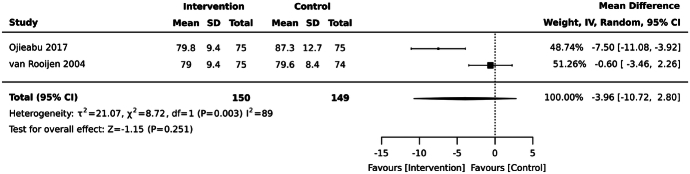
Effect of self-management interventions on diastolic blood pressure (mmHg) at 3 months in adults with type 2 diabetes mellitus in sub-Saharan Africa

**Figure S9:**
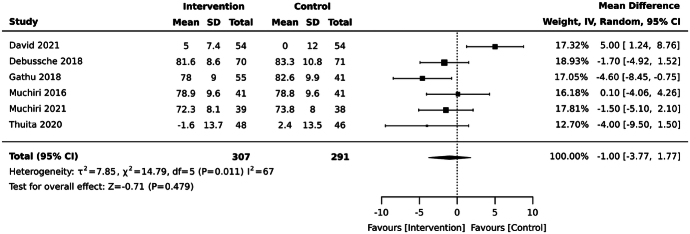
Effect of self-management interventions on diastolic blood pressure (mmHg) at 6 months in adults with type 2 diabetes mellitus in sub-Saharan Africa

**Figure S10:**
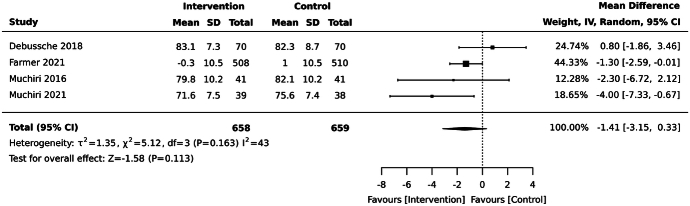
Effect of self-management interventions on diastolic blood pressure (mmHg) at 12 months in adults with type 2 diabetes mellitus in sub-Saharan Africa

**Figure S11:**
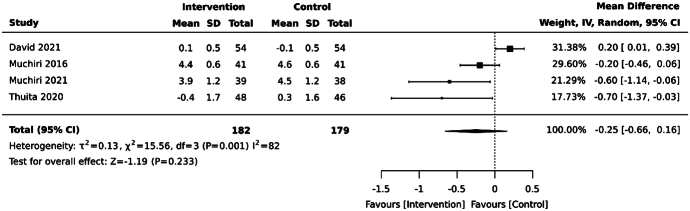
Effect of self-management interventions on total cholesterol (mmol/L) at 6 months in adults with type 2 diabetes mellitus in sub-Saharan Africa

**Figure S12:**
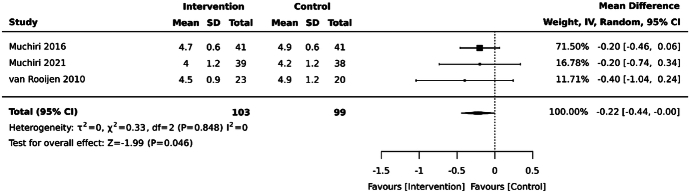
Effect of self-management interventions on total cholesterol (mmol/L) at 12 months in adults with type 2 diabetes mellitus in sub-Saharan Africa

**Figure S13:**
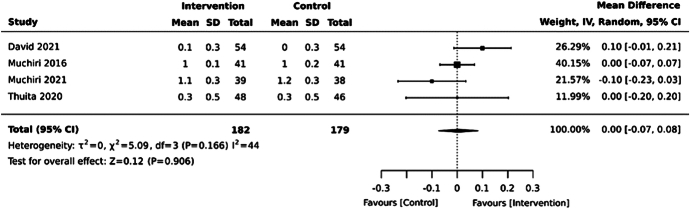
Effect of self-management interventions on high-density lipoprotein (mmol/L) at 6 months in adults with type 2 diabetes mellitus in sub-Saharan Africa

**Figure S14:**
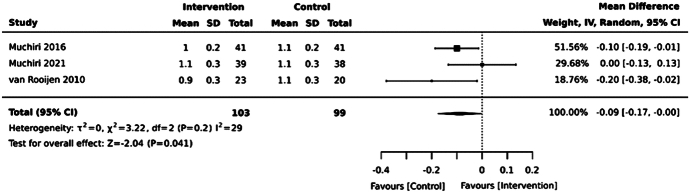
Effect of self-management interventions on high-density lipoprotein (mmol/L) at 12 months in adults with type 2 diabetes mellitus in sub-Saharan Africa

**Figure S15:**
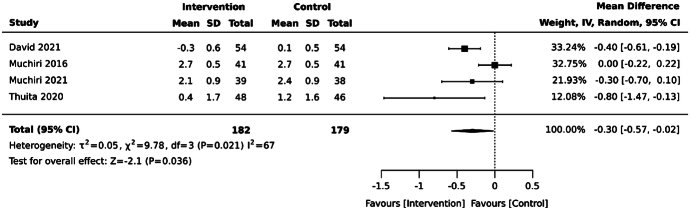
Effect of self-management interventions on low-density lipoprotein (mmol/L) at 6 months in adults with type 2 diabetes mellitus in sub-Saharan Africa

**Figure S16:**
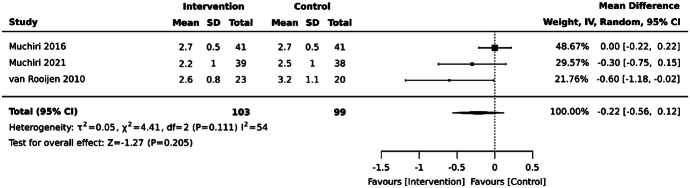
Effect of self-management interventions on low-density lipoprotein (mmol/L) at 12 months in adults with type 2 diabetes mellitus in sub-Saharan Africa

**Figure S17:**
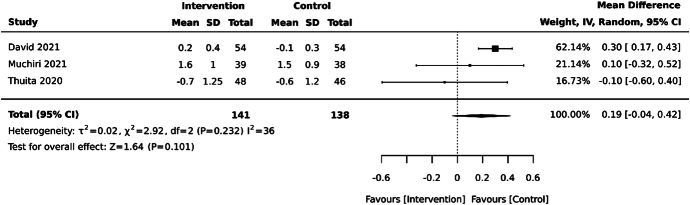
Effect of self-management interventions on trigylcerides (mmol/L) at 6 months in adults with type 2 diabetes mellitus in sub-Saharan Africa

**Figure S18:**
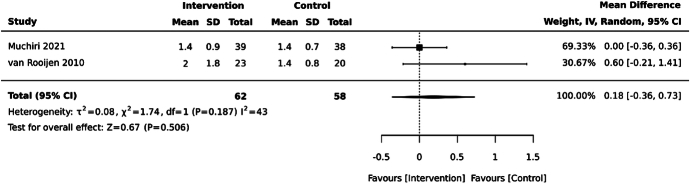
Effect of self-management interventions on triglycerides (mmol/L) at 12 months in adults with type 2 diabetes mellitus in sub-Saharan Africa

**Figure S19:**
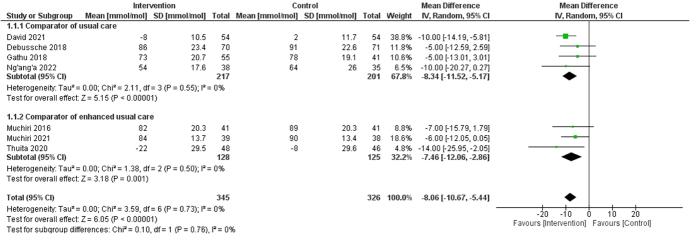
Subgroup analysis of the effect of usual care comparator vs enhanced usual care comparator on HbA1c (mmol/mol) at 6 months in adults with type 2 diabetes mellitus in sub-Saharan Africa

**Figure S20:**
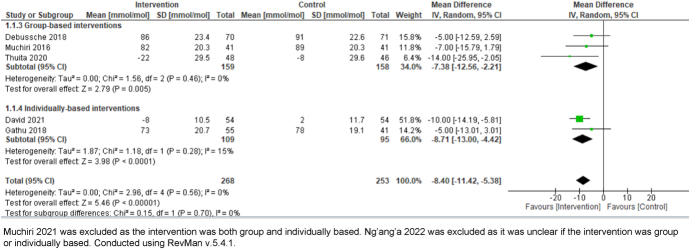
Subgroup analysis of the effect of group-based vs individually based self-management interventions on HbA1c (mmol/mol) at 6 months in adults with type 2 diabetes mellitus in sub-Saharan Africa

**Figure S21:**
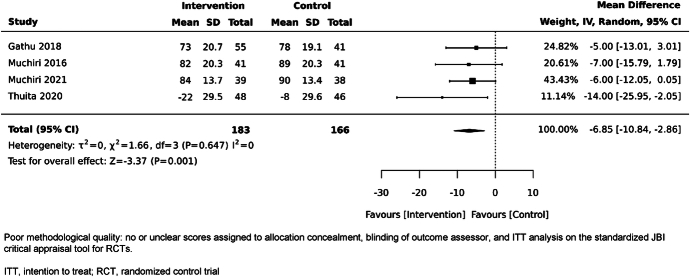
Effect of self-management interventions on HbA1c (mmol/mol) at 6 months in adults with type 2 diabetes mellitus in sub-Saharan Africa: sensitivity analysis with studies of poor methodological quality removed

**Figure S22:**
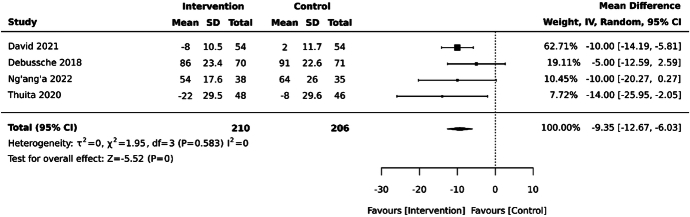
Effect of self-management interventions on HbA1c (mmol/mol) at 6 months in adults with type 2 diabetes mellitus in sub-Saharan Africa: sensitivity analysis with studies with commercial funding removed

**Figure S23:**
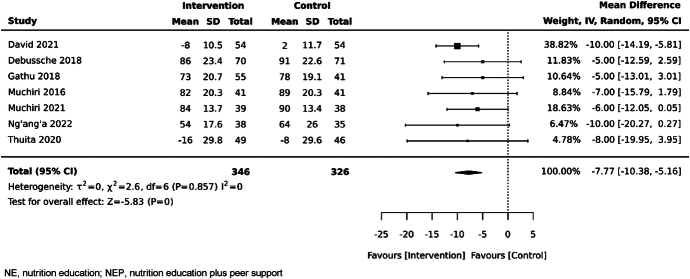
Effect of self-management interventions on HbA1c (mmol/mol) at 6 months in adults with type 2 diabetes mellitus in sub-Saharan Africa: sensitivity analysis with NE arm instead of NEP arm from Thuita 2020

**Figure S24:**
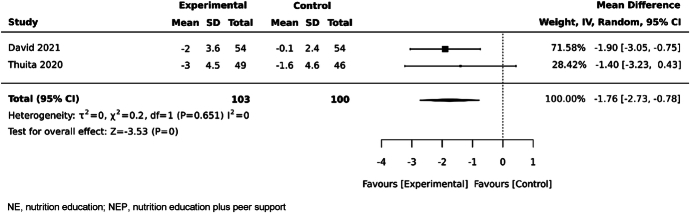
Effect of self-management interventions on fasting blood glucose (mmol/L) at 6 months in adults with type 2 diabetes mellitus in sub-Saharan Africa: sensitivity analysis with NE arm instead of NEP arm from Thuita 2020

**Figure S25:**
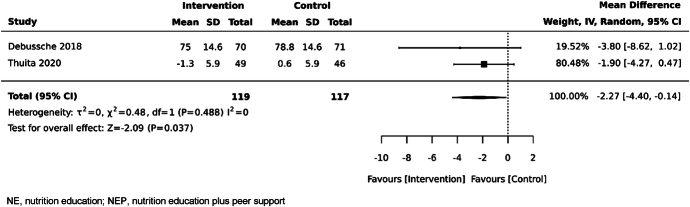
Effect of self-management interventions on weight (kg) at 6 months in adults with type 2 diabetes mellitus in sub-Saharan Africa : sensitivity analysis with NE arm instead of NEP arm from Thuita 2020

**Figure S26:**
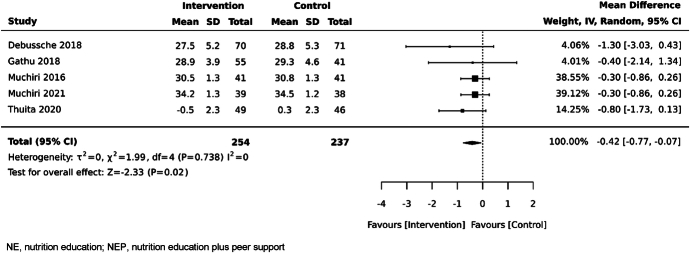
Effect of self-management interventions on body mass index (kg/m^2^) at 6 months in adults with type 2 diabetes mellitus in sub-Saharan Africa: sensitivity analysis with NE arm instead of NEP arm from Thuita 2020

**Figure S27:**
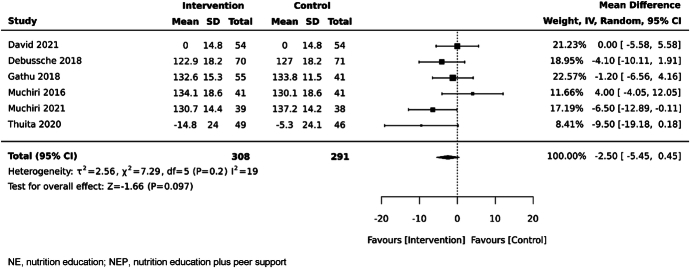
Effect of self-management interventions on systolic blood pressure (mmHg) at 6 months in adults with type 2 diabetes mellitus in sub-Saharan Africa: sensitivity analysis with NE arm instead of NEP arm from Thuita 2020

**Figure S28:**
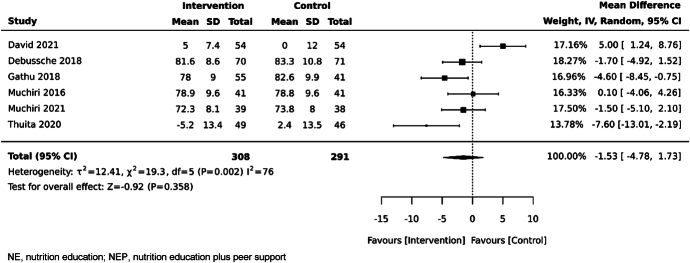
Effect of self-management interventions on diastolic blood pressure (mmHg) at 6 months in adults with type 2 diabetes mellitus in sub-Saharan Africa: sensitivity analysis with NE arm instead of NEP arm from Thuita 2020

**Figure S29:**
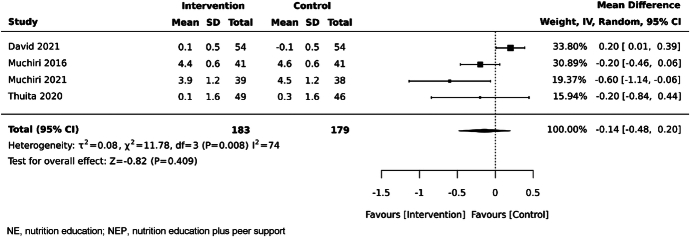
Effect of self-management interventions on total cholesterol (mmol/L) at 6 months in adults with type 2 diabetes mellitus in sub-Saharan Africa: sensitivity analysis with NE arm instead of NEP arm from Thuita 2020

**Figure S30:**
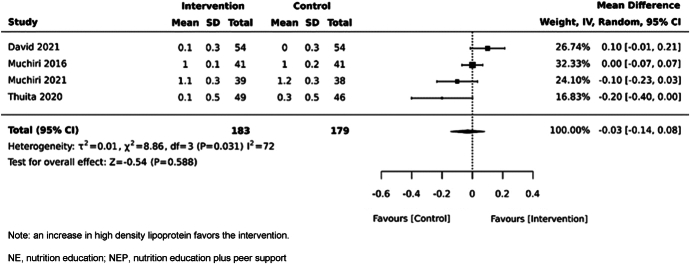
Effect of self-management interventions on high-density lipoprotein (mmol/L) at 6 months in adults with type 2 diabetes mellitus in sub-Saharan Africa: sensitivity analysis with NE arm instead of NEP arm from Thuita 2020

**Figure S31:**
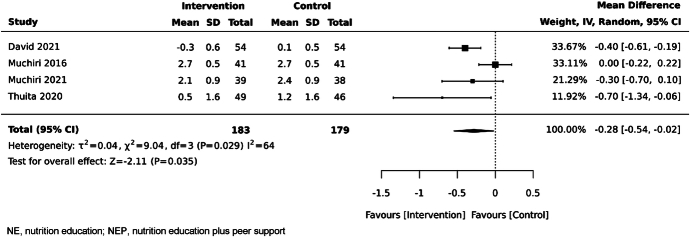
Effect of self-management interventions on low-density lipoprotein (mmol/L) at 6 months in adults with type 2 diabetes mellitus in sub-Saharan Africa: sensitivity analysis with NE arm instead of NEP arm from Thuita 2020

**Figure S32:**
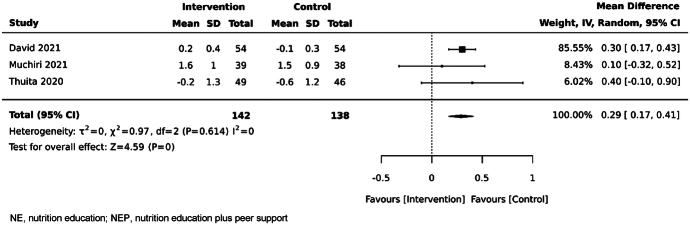
Effect of self-management interventions on trigylcerides (mmol/L) at 6 months in adults with type 2 diabetes mellitus in sub-Saharan Africa: sensitivity analysis with NE arm instead of NEP arm from Thuita 2020

## Appendix I: Search strategy

### MEDLINE (Ovid)

Date searched: January 14, 2023

**Table:**

Search	Search terms	Records retrieved
1	exp Diabetes Mellitus, Type 2/	165,399
2	(type* adj3 (‘2’ or ‘II’ or two*) adj3 (diabete* or diabetic*)).tw.	181,898
3	((late or maturit* or adult* or slow*) adj3 onset* adj3 (diabete* or diabetic*)).tw.	3625
4	((‘ketosis-resistant*‘ or stable*) adj3 (diabete* or diabetic*)).tw.	866
5	((‘non insulin’* or noninsulin*) adj3 depend* adj3 (diabete* or diabetic*)).tw.	11,901
6	(NIDDM or T2DM or T2D).tw.	52,017
7	1 or 2 or 3 or 4 or 5 or 6	236,555
8	exp “Africa South of the Sahara”/	248,889
9	((central or eastern or western or southern) adj1 africa*).tw.	13,988
10	((subsahara* or ‘sub sahara*‘) adj2 africa*).tw.	31,178
11	(Angola or Benin or Botswana or ‘Burkina Faso’ or Burundi or ‘Cabo Verde’ or ‘Cape Verde’ or Cameroon or ‘Central African Republic’ or Chad or Comoros or ‘Democratic Republic of Congo’ or ‘Republic of Congo’ or Congo or ‘Cote Divoire’ or ‘Ivory Coast’ or ‘Equatorial Guinea’ or Eritrea or Eswatini or Swaziland or Ethiopia or Gabon or Gambia or Ghana or Guinea or ‘Guinea-Bissau’ or Kenya or Lesotho or Liberia or Madagascar or Malawi or Mali or Mauritania or Mauritius or Mozambique or Namibia or Niger or Nigeria or Rwanda or “Sao Tome” or Senegal or Seychelles or ‘Sierra Leone’ or Somalia or ‘South Africa’ or ‘South Sudan’ or Sudan or Tanzania or Togo or Uganda or Zambia or Zimbabwe).tw.	372,404
12	8 or 9 or 10 or 11	460,758
13	exp Life Style/	108,951
14	exp Health Education/	260,680
15	exp Exercise/	240,276
16	((Physical fitness/ or physical education.mp.) and training/) or physical exertion/ [mp=title, book title, abstract, original title, name of substance word, subject heading word, floating sub-heading word, keyword heading word, organism supplementary concept word, protocol supplementary concept word, rare disease supplementary concept word, unique identifier, synonyms]	57,404
17	exp Diet/	322,534
18	exp Nutrition Therapy/	112,381
19	'nutrition assessment’/	0
20	Food/	37,412
21	exp Meals/ or exp Dietary Carbohydrates/	106,988
22	(lifestyle* or ‘life style*‘ or educat* or knowledge or exercise* or fitness or ‘physical activit*‘ or diet* or nutrition or food*).tw.	3,078,497
23	(‘non-pharmacolog*‘ or ‘nonpharmocolog’).tw.	13,416
24	exp Patient Education as Topic/	88,492
25	exp Self-Management/	5036
26	exp Self Care/	61,298
27	(‘self management’ or ‘self help’ or ‘self care’ or confidence or ‘self efficacy’ or responsib* or autonomy*).tw.	1,327,565
28	exp Telemedicine/	42,885
29	((tele adj2 (health or medicine or care)) or telehealth or telemedicine or telecare).tw.	26,691
30	exp Cell Phone/	21,567
31	(‘m-health’ or mhealth or ‘mobile health’ or SMS or messag* or ‘mobile phone*‘).tw.	97,225
32	exp Self-Help Groups/ or exp Peer Group/ or exp Social Support/	108,245
33	((peer or patient or emotional or social or psychosocial) adj1 (support or group*)).tw.	145,165
34	exp Medication Adherence/	25,292
35	(monitor* or ‘self-monitor*‘ or selfmonitor*).tw.	953,586
36	((home or environment* or living or assistive) adj2 (adapt* or modif* or equipment or technolog*)).tw.	20,613
37	13 or 14 or 15 or 16 or 17 or 18 or 19 or 20 or 21 or 22 or 23 or 24 or 25 or 26 or 27 or 28 or 29 or 30 or 31 or 32 or 33 or 34 or 35 or 36	5,652,182
38	Randomized Controlled Trial.pt.	584,520
39	Controlled Clinical Trial.pt.	95,157
40	Clinical Trial.pt.	536,836
41	exp Clinical Trials as Topic/	379,683
42	Placebos/	35,924
43	Random Allocation/	106,899
44	Double-Blind Method/	174,010
45	Single-Blind Method/	32,425
46	Cross-Over Studies/	54,551
47	((random$ or control$ or clinical$) adj3 (trial$ or stud$)).tw.	1,510,030
48	(random$ adj3 allocat$).tw.	43,902
49	placebo$.tw.	242,466
50	((singl$ or doubl$ or trebl$ or tripl$) adj (blind$ or mask$)).tw.	194,039
51	(crossover$ or (cross adj over$)).tw.	98,073
52	'field trial’.tw.	4579
53	38 or 39 or 40 or 41 or 42 or 43 or 44 or 45 or 46 or 47 or 48 or 49 or 50 or 51 or 52	2,352,098
54	7 and 12 and 37 and 53	153

### PubMed

Date searched: January 14, 2023

**Table:**

Search	Search terms	Records retrieved
1	“Diabetes Mellitus, Type 2”[Mesh]	165,241
2	(type*[tiab] AND (‘2’[tiab] OR ‘II’[tiab] OR two*[tiab]) AND (diabete*[tiab] OR diabetic*[tiab]))	235,084
3	((late[tiab] OR maturit*[tiab] OR adult*[tiab] OR slow*[tiab]) AND onset*[tiab] AND (diabete*[tiab] OR diabetic*[tiab]))	10,042
4	((‘ketosis-resistant*’[tiab] OR stable*[tiab]) AND (diabete*[tiab] OR diabetic*[tiab]))	13,230
5	((“non insulin*“[tiab] OR noninsulin*[tiab]) AND depend*[tiab] AND (diabete*[tiab] OR diabetic*[tiab]))	12,526
6	(NIDDM[tiab] OR T2DM[tiab] OR T2D[tiab])	52,635
7	#1 OR #2 OR #3 OR #4 OR #5 OR #6	296,413
8	”“Africa South of the Sahara”“[Mesh]	252,958
9	((central[tiab] OR eastern[tiab] OR western[tiab] OR southern[tiab]) AND africa*[tiab])	50,153
10	((subsahara*[tiab] OR “sub sahara*“[tiab]) AND africa*[tiab])	33,624
11	(Angola[tiab] OR Benin[tiab] OR Botswana[tiab] OR “‘Burkina Faso’“[tiab] OR Burundi[tiab] OR “‘Cabo Verde’“[tiab] OR “‘Cape Verde’“[tiab] OR Cameroon[tiab] OR “‘Central African Republic’“[tiab] OR Chad[tiab] OR Comoros[tiab] OR “‘Democratic Republic of Congo’“[tiab] OR “‘Republic of Congo’“[tiab] OR Congo[tiab] OR “‘Cote Divoire’“[tiab] OR “‘Ivory Coast’“[tiab] OR “‘Equatorial Guinea’“[tiab] OR Eritrea[tiab] OR Eswatini[tiab] OR Swaziland[tiab] OR Ethiopia[tiab] OR Gabon[tiab] OR Gambia[tiab] OR Ghana[tiab] OR Guinea[tiab] OR ‘Guinea-Bissau’[tiab] OR Kenya[tiab] OR Lesotho[tiab] OR Liberia[tiab] OR Madagascar[tiab] OR Malawi[tiab] OR Mali[tiab] OR Mauritania[tiab] OR Mauritius[tiab] OR Mozambique[tiab] OR Namibia[tiab] OR Niger[tiab] OR Nigeria[tiab] OR Rwanda[tiab] OR “‘Sao Tome’“[tiab] OR Senegal[tiab] OR Seychelles[tiab] OR “‘Sierra Leone’“[tiab] OR Somalia[tiab] OR “‘South Africa’“[tiab] OR “‘South Sudan’“[tiab] OR Sudan[tiab] OR Tanzania[tiab] OR Togo[tiab] OR Uganda[tiab] OR Zambia[tiab] OR Zimbabwe[tiab])	379,355
12	#8 OR #9 OR #10 OR #11	480,543
13	“Life Style”[Mesh]	108,932
14	“Health Education”[Mesh]	260,653
15	Exercise[Mesh]	240,139
16	”‘Physical fitness’“[Mesh:NoExp] OR “‘physical education” AND training’[Mesh:NoExp] OR “‘physical exertion’“[Mesh:NoExp]	57,526
17	Diet[Mesh]	322,426
18	“Nutrition Therapy”[Mesh]	112,354
19	”‘nutrition assessment’“[Mesh:NoExp]	17,470
20	Food[Mesh:NoExp]	37,398
21	Meals[Mesh] OR “Dietary Carbohydrates”[Mesh]	106,975
22	(lifestyle*[tiab] OR “life style*“[tiab] OR educat*[tiab] OR knowledge[tiab] OR exercise*[tiab] OR fitness[tiab] OR “physical activit*“[tiab] OR diet*[tiab] OR nutrition[tiab] OR food*[tiab])	3,152,441
23	(‘non-pharmacolog*’[tiab] OR ‘nonpharmocolog’[tiab]) - Schema: all - Schema: all - Schema: all	0
24	(‘non-pharmacolog*’[tiab] OR ‘nonpharmocolog’[tiab]) - Schema: all - Schema: all	0
25	“Patient Education as Topic”[Mesh]	88,493
26	Self-Management[Mesh]	5033
27	“Self Care”[Mesh]	61,275
28	(“‘self management’“[tiab] OR “‘self help’“[tiab] OR “‘self care’“[tiab] OR confidence[tiab] OR “‘self efficacy’“[tiab] OR responsib*[tiab] OR autonomy*[tiab])	1,332,805
29	Telemedicine[Mesh]	43,059
30	((tele[tiab] AND (health[tiab] OR medicine[tiab] OR care[tiab])) OR telehealth[tiab] OR telemedicine[tiab] OR telecare[tiab])	33,555
31	“Cell Phone”[Mesh]	21,542
32	(‘m-health’[tiab] OR mhealth[tiab] OR “‘mobile health’“[tiab] OR SMS[tiab] OR messag*[tiab] OR “‘mobile phone*’“[tiab])	88,967
33	“Self-Help Groups”[Mesh] OR “Peer Group”[Mesh] OR “Social Support”[Mesh]	108,233
34	((peer[tiab] OR patient[tiab] OR emotional[tiab] OR social[tiab] OR psychosocial[tiab]) AND (support[tiab] OR group*[tiab]))	954,971
35	“Medication Adherence”[Mesh]	25,281
36	(monitor*[tiab] OR ‘self-monitor*’[tiab] OR selfmonitor*[tiab])	963,719
37	((home[tiab] OR environment*[tiab] OR living[tiab] OR assistive[tiab]) AND (adapt*[tiab] OR modif*[tiab] OR equipment[tiab] OR technolog*[tiab]))	317,039
38	#13 OR #14 OR #15 OR #16 OR #17 OR #18 OR #19 OR #20 OR #21 OR #22 OR #23 OR #24 OR #25 OR #26 OR #27 OR #28 OR #29 OR #30 OR #31 OR #32 OR #33 OR #34 OR #35 OR #36 OR #37	6,417,445
39	“Randomized Controlled Trial”[pt]	585,800
40	“Controlled Clinical Trial”[pt]	676,055
41	“Clinical Trial”[pt]	959,899
42	“Clinical Trials as Topic”[Mesh]	379,574
43	Placebos[Mesh:NoExp]	35,924
44	“Random Allocation”[Mesh:NoExp]	106,898
45	“Double-Blind Method”[Mesh:NoExp]	173,987
46	“Single-Blind Method”[Mesh:NoExp]	32,416
47	“Cross-Over Studies”[Mesh:NoExp]	54,543
48	((random*[tiab] OR control*[tiab] OR clinical*[tiab]) AND (trial*[tiab] OR stud*[tiab]))	5,382,762
49	(random*[tiab] AND allocat*[tiab])	67,653
50	placebo*[tiab]	243,424
51	((singl*[tiab] OR doubl*[tiab] OR trebl*[tiab] OR tripl*[tiab]) AND (blind*[tiab] OR mask*[tiab])) (crossover*[tiab] OR (cross[tiab] AND over*[tiab]))	31,490
52	”‘field trial’“[tiab]	4759
53	#39 OR #40 OR #41 OR #42 OR #43 OR #44 OR #45 OR #46 OR #47 OR #48 OR #49 OR #50 OR #51 OR #52	5,906,528
54	#7 AND #12 AND #38 AND #53	832
55	publisher[sb] or pubmednotmedline[sb]	5,036,549
56	#54 AND #55	200

### Embase (Ovid)

Date searched: January 14, 2023

**Table:**

Search	Search terms	Records retrieved
1	exp non insulin dependent diabetes mellitus/	315,484
2	(type* adj3 (‘2’ or ‘II’ or two*) adj3 (diabete* or diabetic*)).tw.	279,055
3	((late or maturit* or adult* or slow*) adj3 onset* adj3 (diabete* or diabetic*)).tw.	4912
4	((‘ketosis-resistant*‘ or stable*) adj3 (diabete* or diabetic*)).tw.	1348
5	((non insulin* or noninsulin*) adj3 depend* adj3 (diabete* or diabetic*)).tw.	13,850
6	(NIDDM or T2DM or T2D).tw.	85,504
7	1 or 2 or 3 or 4 or 5 or 6	383,823
8	exp “Africa south of the Sahara”/	296,367
9	((central or eastern or western or southern) adj1 africa*).tw.	15,075
10	((subsahara* or sahara*) adj2 africa*).tw.	37,039
11	(Angola or Benin or Botswana or ‘Burkina Faso’ or Burundi or ‘Cabo Verde’ or ‘Cape Verde’ or Cameroon or ‘Central African Republic’ or Chad or Comoros or ‘Democratic Republic of Congo’ or ‘Republic of Congo’ or Congo or ‘Cote Divoire’ or ‘Ivory Coast’ or ‘Equatorial Guinea’ or Eritrea or Eswatini or Swaziland or Ethiopia or Gabon or Gambia or Ghana or Guinea or ‘Guinea-Bissau’ or Kenya or Lesotho or Liberia or Madagascar or Malawi or Mali or Mauritania or Mauritius or Mozambique or Namibia or Niger or Nigeria or Rwanda or ‘Sao Tome’ or Senegal or Seychelles or ‘Sierra Leone’ or Somalia or ‘South Africa’ or ‘South Sudan’ or Sudan or Tanzania or Togo or Uganda or Zambia or Zimbabwe).tw.	410,983
12	8 or 9 or 10 or 11	490,112
13	exp lifestyle/ or exp lifestyle modification/	200,869
14	exp health education/	365,640
15	exp fitness/	41,156
16	((‘Physical fitness’/ or ‘physical education.mp.) and training’/) or ‘physical exertion’/ [mp=title, abstract, heading word, drug trade name, original title, device manufacturer, drug manufacturer, device trade name, keyword heading word, floating subheading word, candidate term word]	297,413
17	exp diet/	382,648
18	exp nutrition/	2,540,835
19	exp food/	1,194,211
20	exp meal/	25,593
21	exp carbohydrate intake/	32,648
22	(lifestyle* or life style* or educat* or knowledge or exercise* or fitness or physical activit* or diet* or nutrition or food*).tw.	3,845,661
23	(‘non-pharmacolog*‘ or ‘nonpharmocolog’).tw.	21,254
24	exp patient education/	122,817
25	exp self care/	98,114
26	(‘self management’ or ‘self help’ or ‘self care’ or confidence or ‘self efficacy’ or responsib* or autonomy*).tw.	1,657,129
27	exp telemedicine/	65,593
28	((tele adj2 (health or medicine or care)) or telehealth or telemedicine or telecare).tw.	36,451
29	exp mobile phone/	43,693
30	(‘m-health’ or mhealth or ‘mobile health’ or SMS or messag* or ‘mobile phone*‘).tw.	124,073
31	exp self help/	14,497
32	exp peer group/	29,299
33	exp social support/	111,058
34	((peer or patient or emotional or social or psychosocial) adj1 (support or group*)).tw.	203,034
35	exp medication compliance/	43,415
36	((home or environment* or living or assistive) adj2 (adapt* or modif* or equipment or technolog*)).tw.	24,103
37	13 or 14 or 15 or 16 or 17 or 18 or 19 or 20 or 21 or 22 or 23 or 24 or 25 or 26 or 27 or 28 or 29 or 30 or 31 or 32 or 33 or 34 or 35 or 36	7,204,924
38	exp randomized controlled trial/	747,120
39	exp clinical trial/	1,766,345
40	exp placebo/	390,562
41	exp randomization/	96,289
42	exp controlled study/ or exp double blind procedure/	9,526,647
43	exp single blind procedure/	48,926
44	exp crossover procedure/	72,662
45	((random$ or control$ or clinical$) adj3 (trial$ or stud$)).tw.	2,096,678
46	(random$ adj3 allocat$).tw.	54,354
47	placebo$.tw.	354,608
48	((singl$ or doubl$ or trebl$ or tripl$) adj (blind$ or mask$)).tw.	272,328
49	(crossover$ or (cross adj over$)).tw.	121,925
50	'field trial’.tw.	4486
51	38 or 39 or 40 or 41 or 42 or 43 or 44 or 45 or 46 or 47 or 48 or 49 or 50	11,204,521
52	7 and 12 and 37 and 51	771

### CINAHL (EBSCOhost)

Date searched: January 14, 2023

**Table:**

Search	Search terms	Records retrieved
1	(MM “Diabetes Mellitus, Type 2”)	56,008
2	((TI type* OR AB type*) N3 ((TI ‘2’ OR AB ‘2’) OR (TI ‘II’ OR AB ‘II’) OR (TI two* OR AB two*)) N3 ((TI diabete* OR AB diabete*) OR (TI diabetic* OR AB diabetic*)))	65,988
3	(((TI late OR AB late) OR (TI maturit* OR AB maturit*) OR (TI adult* OR AB adult*) OR (TI slow* OR AB slow*)) N3 (TI onset* OR AB onset*) N3 ((TI diabete* OR AB diabete*) OR (TI diabetic* OR AB diabetic*)))	949
4	(((TI ‘ketosis-resistant*’ OR AB ‘ketosis-resistant*’) OR (TI stable* OR AB stable*)) N3 ((TI diabete* OR AB diabete*) OR (TI diabetic* OR AB diabetic*)))	299
5	(((TI “non insulin*“ OR AB “non insulin*“) OR (TI noninsulin* OR AB noninsulin*)) N3 (TI depend* OR AB depend*) N3 ((TI diabete* OR AB diabete*) OR (TI diabetic* OR AB diabetic*)))	1529
6	((TI NIDDM OR AB NIDDM) OR (TI T2DM OR AB T2DM) OR (TI T2D OR AB T2D))	14,173
7	S1 OR S2 OR S3 OR S4 OR S5 OR S6	83,067
8	(MH “Africa South of the Sahara+“)	83,118
9	(((TI central OR AB central) OR (TI eastern OR AB eastern) OR (TI western OR AB western) OR (TI southern OR AB southern)) N1 (TI africa* OR AB africa*))	2590
10	(((TI subsahara* OR AB subsahara*) OR (TI “sub sahara*“ OR AB “sub sahara*“)) N2 (TI africa* OR AB africa*))	10,011
11	((TI Angola OR AB Angola) OR (TI Benin OR AB Benin) OR (TI Botswana OR AB Botswana) OR (TI “‘Burkina Faso’“ OR AB “‘Burkina Faso’“) OR (TI Burundi OR AB Burundi) OR (TI “‘Cabo Verde’“ OR AB “‘Cabo Verde’“) OR (TI “‘Cape Verde’“ OR AB “‘Cape Verde’“) OR (TI Cameroon OR AB Cameroon) OR (TI “‘Central African Republic’“ OR AB “‘Central African Republic’“) OR (TI Chad OR AB Chad) OR (TI Comoros OR AB Comoros) OR (TI “‘Democratic Republic of Congo’“ OR AB “‘Democratic Republic of Congo’“) OR (TI “‘Republic of Congo’“ OR AB “‘Republic of Congo’“) OR (TI Congo OR AB Congo) OR (TI “‘Cote Divoire’“ OR AB “‘Cote Divoire’“) OR (TI “‘Ivory Coast’“ OR AB “‘Ivory Coast’“) OR (TI “‘Equatorial Guinea’“ OR AB “‘Equatorial Guinea’“) OR (TI Eritrea OR AB Eritrea) OR (TI Eswatini OR AB Eswatini) OR (TI Swaziland OR AB Swaziland) OR (TI Ethiopia OR AB Ethiopia) OR (TI Gabon OR AB Gabon) OR (TI Gambia OR AB Gambia) OR (TI Ghana OR AB Ghana) OR (TI Guinea OR AB Guinea) OR (TI ‘Guinea-Bissau’ OR AB ‘Guinea-Bissau’) OR (TI Kenya OR AB Kenya) OR (TI Lesotho OR AB Lesotho) OR (TI Liberia OR AB Liberia) OR (TI Madagascar OR AB Madagascar) OR (TI Malawi OR AB Malawi) OR (TI Mali OR AB Mali) OR (TI Mauritania OR AB Mauritania) OR (TI Mauritius OR AB Mauritius) OR (TI Mozambique OR AB Mozambique) OR (TI Namibia OR AB Namibia) OR (TI Niger OR AB Niger) OR (TI Nigeria OR AB Nigeria) OR (TI Rwanda OR AB Rwanda) OR (TI “‘Sao Tome’“ OR AB “‘Sao Tome’“) OR (TI Senegal OR AB Senegal) OR (TI Seychelles OR AB Seychelles) OR (TI “‘Sierra Leone’“ OR AB “‘Sierra Leone’“) OR (TI Somalia OR AB Somalia) OR (TI “‘South Africa’“ OR AB “‘South Africa’“) OR (TI “‘South Sudan’“ OR AB “‘South Sudan’“) OR (TI Sudan OR AB Sudan) OR (TI Tanzania OR AB Tanzania) OR (TI Togo OR AB Togo) OR (TI Uganda OR AB Uganda) OR (TI Zambia OR AB Zambia) OR (TI Zimbabwe OR AB Zimbabwe))	75,265
12	S8 OR S9 OR S10 OR S11	105,312
13	(MH “Life Style+“)	271,240
14	(MH “Health Education+“)	141,688
15	(MH “Exercise+“)	130,387
16	(MH “Physical Fitness+“)	20,967
17	(MH “Diet+“)	141,067
18	(MH “Nutrition+“)	187,587
19	(MH “Food+“)	202,677
20	(MH “Meals+“)	11,575
21	(MH “Dietary Carbohydrates+“)	13,327
22	(MH “Patient Education+“)	85,057
23	(MH “Self-Management”)	2582
24	(MH “Self Care+“)	59,832
25	((TI “‘self management’“ OR AB “‘self management’“) OR (TI “‘self help’“ OR AB “‘self help’“) OR (TI “‘self care’“ OR AB “‘self care’“) OR (TI confidence OR AB confidence) OR (TI “‘self efficacy’“ OR AB “‘self efficacy’“) OR (TI responsib* OR AB responsib*) OR (TI autonomy* OR AB autonomy*))	388,321
26	(MH “Telemedicine+“)	19,318
27	(((TI tele OR AB tele) N2 ((TI health OR AB health) OR (TI medicine OR AB medicine) OR (TI care OR AB care))) OR (TI telehealth OR AB telehealth) OR (TI telemedicine OR AB telemedicine) OR (TI telecare OR AB telecare))	15,680
28	(MH “Cellular Phone+“)	10,031
29	((TI ‘m-health’ OR AB ‘m-health’) OR (TI mhealth OR AB mhealth) OR (TI “‘mobile health’“ OR AB “‘mobile health’“) OR (TI SMS OR AB SMS) OR (TI messag* OR AB messag*) OR (TI “‘mobile phone*’“ OR AB “‘mobile phone*’“))	54,849
30	(MH “Support Groups+“)	12,254
31	(MH “Peer Counseling”)	1140
32	(((TI peer OR AB peer) OR (TI patient OR AB patient) OR (TI emotional OR AB emotional) OR (TI social OR AB social) OR (TI psychosocial OR AB psychosocial)) N1 ((TI support OR AB support) OR (TI group* OR AB group*)))	134,657
33	(MH “Medication Compliance”)	23,384
34	((TI monitor* OR AB monitor*) OR (TI ‘self-monitor*’ OR AB ‘self-monitor*’) OR (TI selfmonitor* OR AB selfmonitor*))	181,634
35	(((TI home OR AB home) OR (TI environment* OR AB environment*) OR (TI living OR AB living) OR (TI assistive OR AB assistive)) N2 ((TI adapt* OR AB adapt*) OR (TI modif* OR AB modif*) OR (TI equipment OR AB equipment) OR (TI technolog* OR AB technolog*)))	10,008
36	S13 OR S14 OR S15 OR S16 OR S17 OR S18 OR S19 OR S20 OR S21 OR S22 OR S23 OR S24 OR S25 OR S26 OR S27 OR S28 OR S29 OR S30 OR S31 OR S32 OR S33 OR S34 OR S35	1,488,584
37	PT “Randomized Controlled Trial”	149,591
38	PT “Clinical Trial”	114,475
39	(MH “Randomized Controlled Trials+“) OR (MH “Clinical Trials+“)	348,444
40	(MH “Placebos”)	13,925
41	(MH “Random Assignment”)	77,466
42	(MH “Double-Blind Studies”) OR (MH “Single-Blind Studies”)	69,664
43	(MH “Crossover Design”)	21,705
44	(((TI random* OR AB random*) OR (TI control* OR AB control*) OR (TI clinical* OR AB clinical*)) N3 ((TI trial* OR AB trial*) OR (TI stud* OR AB stud*)))	511,950
45	((TI random* OR AB random*) N3 (TI allocat* OR AB allocat*))	14,893
46	(TI placebo* OR AB placebo*)	72,572
47	(((TI singl* OR AB singl*) OR (TI doubl* OR AB doubl*) OR (TI trebl* OR AB trebl*) OR (TI tripl* OR AB tripl*)) W1 ((TI blind* OR AB blind*) OR (TI mask* OR AB mask*)))	58,062
48	((TI crossover* OR AB crossover*) OR ((TI cross OR AB cross) W1 (TI over* OR AB over*)))	23,254
49	(TI “‘field trial’“ OR AB “‘field trial’“)	521
50	S37 OR S38 OR S39 OR S40 OR S41 OR S42 OR S43 OR S44 OR S45 OR S46 OR S47 OR S48 OR S49	716,988
51	S7 AND S12 AND S36 AND S50	52

### PsycINFO (Ovid)

Date searched: January 14, 2023

**Table:**

Search	Search terms	Records retrieved
1	exp type 2 diabetes/	5612
2	(type* adj3 (‘2’ or ‘II’ or two*) adj3 (diabete* or diabetic*)).ti,ab.	9485
3	((late or maturit* or adult* or slow*) adj3 onset* adj3 (diabete* or diabetic*)).ti,ab.	89
4	((‘ketosis-resistant*‘ or stable*) adj3 (diabete* or diabetic*)).ti,ab.	21
5	((“non insulin*“ or noninsulin*) adj3 depend* adj3 (diabete* or diabetic*)).ti,ab.	263
6	(NIDDM or T2DM or T2D).ti,ab.	2052
7	1 or 2 or 3 or 4 or 5 or 6	11,024
8	“Africa South of the Sahara”.mp. [mp=title, abstract, heading word, table of contents, key concepts, original title, tests & measures, mesh word]	669
9	((central or eastern or western or southern) adj1 africa*).ti,ab.	1500
10	((subsahara* or “sub sahara*“) adj2 africa*).ti,ab.	4733
11	(Angola or Benin or Botswana or “Burkina Faso” or Burundi or “Cabo Verde” or “Cape Verde” or Cameroon or “Central African Republic” or Chad or Comoros or “Democratic Republic of Congo” or “Republic of Congo” or Congo or “Cote Divoire” or “Ivory Coast” or “Equatorial Guinea” or Eritrea or Eswatini or Swaziland or Ethiopia or Gabon or Gambia or Ghana or Guinea or “Guinea-Bissau” or Kenya or Lesotho or Liberia or Madagascar or Malawi or Mali or Mauritania or Mauritius or Mozambique or Namibia or Niger or Nigeria or Rwanda or “Sao Tome” or Senegal or Seychelles or “Sierra Leone” or Somalia or “South Africa” or “South Sudan” or Sudan or Tanzania or Togo or Uganda or Zambia or Zimbabwe).ti,ab.	46,546
12	8 or 9 or 10 or 11	49,351
13	Life Style.mp.	11,975
14	Health Education.mp.	27,947
15	Exercise.mp.	73,720
16	(((“Physical fitness” or “physical education”) and training) or “physical exertion”).mp. [mp=title, abstract, heading word, table of contents, key concepts, original title, tests & measures, mesh word]	6422
17	Diet.mp.	35,109
18	Nutrition Therapy.mp.	198
19	nutrition assessment.mp.	743
20	Food.mp.	101,670
21	(Meals or “Dietary Carbohydrates”).mp.	6668
22	(lifestyle* or “life style*“ or educat* or knowledge or exercise* or fitness or “physical activit*“ or diet* or nutrition or food*).ti,ab.	1,027,356
23	(‘non-pharmacolog*‘ or ‘nonpharmocolog’).ti,ab.	3413
24	Patient Education.mp.	14,206
25	(“self management” or “self help” or “self care” or confidence or “self efficacy” or responsib* or autonomy*).ti,ab.	312,144
26	((tele adj2 (health or medicine or care)) or telehealth or telemedicine or telecare).ti,ab.	4799
27	Cell Phone.mp.	1723
28	(‘m-health’ or mhealth or “‘mobile health’“ or SMS or messag* or “mobile phone*“).ti,ab.	53,698
29	(“Self-Help Groups” or “Peer Group” or “Social Support”).mp.	109,139
30	((peer or patient or emotional or social or psychosocial) adj1 (support or group*)).ti,ab.	96,610
31	Medication Adherence.mp.	7772
32	(monitor* or ‘self-monitor*‘ or selfmonitor*).ti,ab.	102,124
33	((home or environment* or living or assistive) adj2 (adapt* or modif* or equipment or technolog*)).ti,ab.	7291
34	13 or 14 or 15 or 16 or 17 or 18 or 19 or 20 or 21 or 22 or 23 or 24 or 25 or 26 or 27 or 28 or 29 or 30 or 31 or 32 or 33	1,487,130
35	Randomized Controlled Trial.pt.	0
36	Controlled Clinical Trial.pt.	0
37	exp clinical trials/	13,439
38	Placebos.mp.	6019
39	Random Allocation.mp.	8499
40	Double-Blind Method.mp.	19,382
41	Single-Blind Method.mp.	3097
42	Cross-Over Studies.mp.	5378
43	((random* or control* or clinical*) adj3 (trial* or stud*)).ti,ab.	189,810
44	(random* adj3 allocat*).ti,ab.	5074
45	placebo*.ti,ab.	43,322
46	((singl* or doubl* or trebl* or tripl*) adj (blind* or mask*)).ti,ab.	28,553
47	(crossover* or (cross adj over*)).ti,ab.	11,586
48	field trial.ti,ab.	623
49	35 or 36 or 37 or 38 or 39 or 40 or 41 or 42 or 43 or 44 or 45 or 46 or 47 or 48	235,703
50	7 and 12 and 34 and 49	9

### Scopus

Date searched: January 14, 2023

**Table:**

Search	Search terms	Records retrieved
1	( INDEXTERMS (“Diabetes Mellitus, Type 2”) OR (TITLE-ABS (“type*”) W/3 (TITLE-ABS (“‘2’”) OR TITLE-ABS (“‘II’”) OR TITLE-ABS (“two*”)) W/3 (TITLE-ABS (“diabete*”) OR TITLE-ABS (“diabetic*”))) OR ((TITLE-ABS (“late”) OR TITLE-ABS (“maturit*”) OR TITLE-ABS (“adult*”) OR TITLE-ABS (“slow*”)) W/3 TITLE-ABS (“onset*”) W/3 (TITLE-ABS (“diabete*”) OR TITLE-ABS (“diabetic*”))) OR ((TITLE-ABS (“‘ketosis-resistant*‘”) OR TITLE-ABS (“stable*”)) W/3 (TITLE-ABS (“diabete*”) OR TITLE-ABS (“diabetic*”))) OR ((TITLE-ABS (“non insulin*”) OR TITLE-ABS (“noninsulin*”)) W/3 TITLE-ABS (“depend*”) W/3 (TITLE-ABS (“diabete*”) OR TITLE-ABS (“diabetic*”))) OR (TITLE-ABS (“NIDDM”) OR TITLE-ABS (“T2DM”) OR TITLE-ABS (“T2D”))) AND (INDEXTERMS (“” africa AND south AND of AND the AND sahara “”) OR ((TITLE-ABS (“central”) OR TITLE-ABS (“eastern”) OR TITLE-ABS (“western”) OR TITLE-ABS (“southern”)) W/1 TITLE-ABS (“africa*”)) OR ((TITLE-ABS (“subsahara*”) OR TITLE-ABS (“sub sahara*”)) W/2 TITLE-ABS (“africa*”)) OR (TITLE-ABS (“Angola”) OR TITLE-ABS (“Benin”) OR TITLE-ABS (“Botswana”) OR TITLE-ABS (“‘Burkina Faso’”) OR TITLE-ABS (“Burundi”) OR TITLE-ABS (“‘Cabo Verde’”) OR TITLE-ABS (“‘Cape Verde’”) OR TITLE-ABS (“Cameroon”) OR TITLE-ABS (“‘Central African Republic’”) OR TITLE-ABS (“Chad”) OR TITLE-ABS (“Comoros”) OR TITLE-ABS (“‘Democratic Republic of Congo’”) OR TITLE-ABS (“‘Republic of Congo’”) OR TITLE-ABS (“Congo”) OR TITLE-ABS (“‘Cote Divoire’”) OR TITLE-ABS (“‘Ivory Coast’”) OR TITLE-ABS (“‘Equatorial Guinea’”) OR TITLE-ABS (“Eritrea”) OR TITLE-ABS (“Eswatini”) OR TITLE-ABS (“Swaziland”) OR TITLE-ABS (“Ethiopia”) OR TITLE-ABS (“Gabon”) OR TITLE-ABS (“Gambia”) OR TITLE-ABS (“Ghana”) OR TITLE-ABS (“Guinea”) OR TITLE-ABS (“‘Guinea-Bissau’”) OR TITLE-ABS (“Kenya”) OR TITLE-ABS (“Lesotho”) OR TITLE-ABS (“Liberia”) OR TITLE-ABS (“Madagascar”) OR TITLE-ABS (“Malawi”) OR TITLE-ABS (“Mali”) OR TITLE-ABS (“Mauritania”) OR TITLE-ABS (“Mauritius”) OR TITLE-ABS (“Mozambique”) OR TITLE-ABS (“Namibia”) OR TITLE-ABS (“Niger”) OR TITLE-ABS (“Nigeria”) OR TITLE-ABS (“Rwanda”) OR TITLE-ABS (“‘Sao Tome’”) OR TITLE-ABS (“Senegal”) OR TITLE-ABS (“Seychelles”) OR TITLE-ABS (“‘Sierra Leone’”) OR TITLE-ABS (“Somalia”) OR TITLE-ABS (“‘South Africa’”) OR TITLE-ABS (“‘South Sudan’”) OR TITLE-ABS (“Sudan”) OR TITLE-ABS (“Tanzania”) OR TITLE-ABS (“Togo”) OR TITLE-ABS (“Uganda”) OR TITLE-ABS (“Zambia”) OR TITLE-ABS (“Zimbabwe”))) AND (trial* OR placebo OR control OR controlled OR blind OR random OR randomized OR randomised OR crossover OR “cross over” OR “cross-over”) AND (TITLE-ABS (“Life Style”) OR TITLE-ABS (“Education”) OR TITLE-ABS (“Exercise”) OR TITLE-ABS (“Physical”) OR TITLE-ABS (“Diet”) OR TITLE-ABS (“Nutrition”) OR TITLE-ABS (“Food”) OR TITLE-ABS (“Meals”) OR TITLE-ABS (“Dietary Carbohydrates”) OR TITLE-ABS (“lifestyle”) OR TITLE-ABS (“life style”) OR TITLE-ABS (“knowledge”) OR TITLE-ABS (“fitness”) OR TITLE-ABS (“nonpharmocological”) OR TITLE-ABS (“Self Management”) OR TITLE-ABS (“Self Care”) OR TITLE-ABS (“self help”) OR TITLE-ABS (“confidence”) OR TITLE-ABS (“self efficacy”) OR TITLE-ABS (“responsibility”) OR TITLE-ABS (“autonomy”) OR TITLE-ABS (“Telemedicine”) OR TITLE-ABS (“telehealth”) OR TITLE-ABS (“Cell Phone”) OR TITLE-ABS (“m health”) OR TITLE-ABS (“mhealth”) OR TITLE-ABS (“mobile”) OR TITLE-ABS (“SMS”) OR TITLE-ABS (“messaging”) OR TITLE-ABS (“Peer”) OR TITLE-ABS (“Social Support”) OR TITLE-ABS (“group”) OR TITLE-ABS (“Adherence”) OR TITLE-ABS (“compliance”) OR TITLE-ABS (“self monitor*”))	1180

### EBSCOhost Global Health

Date searched: June 8, 2021

**Table:**

Search	Search terms	Records retrieved
1	(ZU “diabetes mellitus”)	49,369
2	((TI type* OR AB type*) N3 ((TI ‘2’ OR AB ‘2’) OR (TI ‘II’ OR AB ‘II’) OR (TI two* OR AB two*)) N3 ((TI diabete* OR AB diabete*) OR (TI diabetic* OR AB diabetic*)))	38,531
3	(((TI late OR AB late) OR (TI maturit* OR AB maturit*) OR (TI adult* OR AB adult*) OR (TI slow* OR AB slow*)) N3 (TI onset* OR AB onset*) N3 ((TI diabete* OR AB diabete*) OR (TI diabetic* OR AB diabetic*)))	520
4	(((TI ‘ketosis-resistant*’ OR AB ‘ketosis-resistant*’) OR (TI stable* OR AB stable*)) N3 ((TI diabete* OR AB diabete*) OR (TI diabetic* OR AB diabetic*)))	183
5	(((TI “non insulin*“ OR AB “non insulin*“) OR (TI noninsulin* OR AB noninsulin*)) N3 (TI depend* OR AB depend*) N3 ((TI diabete* OR AB diabete*) OR (TI diabetic* OR AB diabetic*)))	1979
6	((TI NIDDM OR AB NIDDM) OR (TI T2DM OR AB T2DM) OR (TI T2D OR AB T2D))	9541
7	S1 OR S2 OR S3 OR S4 OR S5 OR S6	74,659
8	(ZU “africa south of sahara”)	188,051
9	(((TI central OR AB central) OR (TI eastern OR AB eastern) OR (TI western OR AB western) OR (TI southern OR AB southern)) N1 (TI africa* OR AB africa*))	7755
10	(((TI subsahara* OR AB subsahara*) OR (TI “sub sahara*“ OR AB “sub sahara*“)) N2 (TI africa* OR AB africa*))	17,662
11	((TI Angola OR AB Angola) OR (TI Benin OR AB Benin) OR (TI Botswana OR AB Botswana) OR (TI “‘Burkina Faso’“ OR AB “‘Burkina Faso’“) OR (TI Burundi OR AB Burundi) OR (TI “‘Cabo Verde’“ OR AB “‘Cabo Verde’“) OR (TI “‘Cape Verde’“ OR AB “‘Cape Verde’“) OR (TI Cameroon OR AB Cameroon) OR (TI “‘Central African Republic’“ OR AB “‘Central African Republic’“) OR (TI Chad OR AB Chad) OR (TI Comoros OR AB Comoros) OR (TI “‘Democratic Republic of Congo’“ OR AB “‘Democratic Republic of Congo’“) OR (TI “‘Republic of Congo’“ OR AB “‘Republic of Congo’“) OR (TI Congo OR AB Congo) OR (TI “‘Cote Divoire’“ OR AB “‘Cote Divoire’“) OR (TI “‘Ivory Coast’“ OR AB “‘Ivory Coast’“) OR (TI “‘Equatorial Guinea’“ OR AB “‘Equatorial Guinea’“) OR (TI Eritrea OR AB Eritrea) OR (TI Eswatini OR AB Eswatini) OR (TI Swaziland OR AB Swaziland) OR (TI Ethiopia OR AB Ethiopia) OR (TI Gabon OR AB Gabon) OR (TI Gambia OR AB Gambia) OR (TI Ghana OR AB Ghana) OR (TI Guinea OR AB Guinea) OR (TI ‘Guinea-Bissau’ OR AB ‘Guinea-Bissau’) OR (TI Kenya OR AB Kenya) OR (TI Lesotho OR AB Lesotho) OR (TI Liberia OR AB Liberia) OR (TI Madagascar OR AB Madagascar) OR (TI Malawi OR AB Malawi) OR (TI Mali OR AB Mali) OR (TI Mauritania OR AB Mauritania) OR (TI Mauritius OR AB Mauritius) OR (TI Mozambique OR AB Mozambique) OR (TI Namibia OR AB Namibia) OR (TI Niger OR AB Niger) OR (TI Nigeria OR AB Nigeria) OR (TI Rwanda OR AB Rwanda) OR (TI “‘Sao Tome’“ OR AB “‘Sao Tome’“) OR (TI Senegal OR AB Senegal) OR (TI Seychelles OR AB Seychelles) OR (TI “‘Sierra Leone’“ OR AB “‘Sierra Leone’“) OR (TI Somalia OR AB Somalia) OR (TI “‘South Africa’“ OR AB “‘South Africa’“) OR (TI “‘South Sudan’“ OR AB “‘South Sudan’“) OR (TI Sudan OR AB Sudan) OR (TI Tanzania OR AB Tanzania) OR (TI Togo OR AB Togo) OR (TI Uganda OR AB Uganda) OR (TI Zambia OR AB Zambia) OR (TI Zimbabwe OR AB Zimbabwe))	180,700
12	S8 OR S9 OR S10 OR S11	220,769
13	(ZU “lifestyle”) or (ZU “health education”) or (ZU “exercise”) or (ZU “physical activity”) or (ZU “diet”) or (ZU “nutrition”) or (ZU “nutrition education”) or (ZU “food”) or (ZU “meal”) or (ZU “carbohydrates”)	289,634
14	((TI lifestyle* OR AB lifestyle*) OR (TI “life style*“ OR AB “life style*“) OR (TI educat* OR AB educat*) OR (TI knowledge OR AB knowledge) OR (TI exercise* OR AB exercise*) OR (TI fitness OR AB fitness) OR (TI “physical activit*“ OR AB “physical activit*“) OR (TI diet* OR AB diet*) OR (TI nutrition OR AB nutrition) OR (TI food* OR AB food*))	933,883
15	((TI ‘non-pharmacolog*’ OR AB ‘non-pharmacolog*’) OR (TI ‘nonpharmocolog’ OR AB ‘nonpharmocolog’))	1109
16	(ZU “patient education”) or (ZU “self management”) or (ZU “self care”)	2699
17	((TI “‘self management’“ OR AB “‘self management’“) OR (TI “‘self help’“ OR AB “‘self help’“) OR (TI “‘self care’“ OR AB “‘self care’“) OR (TI confidence OR AB confidence) OR (TI “‘self efficacy’“ OR AB “‘self efficacy’“) OR (TI responsib* OR AB responsib*) OR (TI autonomy* OR AB autonomy*))	239,791
18	(ZU “telemedicine”)	1966
19	(((TI tele OR AB tele) N2 ((TI health OR AB health) OR (TI medicine OR AB medicine) OR (TI care OR AB care))) OR (TI telehealth OR AB telehealth) OR (TI telemedicine OR AB telemedicine) OR (TI telecare OR AB telecare))	2004
20	(ZU “mobile telephones”)	3329
21	((TI ‘m-health’ OR AB ‘m-health’) OR (TI mhealth OR AB mhealth) OR (TI “‘mobile health’“ OR AB “‘mobile health’“) OR (TI SMS OR AB SMS) OR (TI messag* OR AB messag*) OR (TI “‘mobile phone*’“ OR AB “‘mobile phone*’“))	16,938
22	(ZU “self help”) or (ZU “support measures”) or (ZU “support systems”)	5574
23	(((TI peer OR AB peer) OR (TI patient OR AB patient) OR (TI emotional OR AB emotional) OR (TI social OR AB social) OR (TI psychosocial OR AB psychosocial)) N1 ((TI support OR AB support) OR (TI group* OR AB group*)))	46,479
24	adherence OR compliance	53,706
25	((TI monitor* OR AB monitor*) OR (TI ‘self-monitor*’ OR AB ‘self-monitor*’) OR (TI selfmonitor* OR AB selfmonitor*))	151,507
26	(((TI home OR AB home) OR (TI environment* OR AB environment*) OR (TI living OR AB living) OR (TI assistive OR AB assistive)) N2 ((TI adapt* OR AB adapt*) OR (TI modif* OR AB modif*) OR (TI equipment OR AB equipment) OR (TI technolog* OR AB technolog*)))	5096
27	S13 OR S14 OR S15 OR S16 OR S17 OR S18 OR S19 OR S20 OR S21 OR S22 OR S23 OR S24 OR S25 OR S26	1,312,167
28	(ZU “randomized controlled trials”)	46,272
29	(((TI random* OR AB random*) OR (TI control* OR AB control*) OR (TI clinical* OR AB clinical*)) N3 ((TI trial* OR AB trial*) OR (TI stud* OR AB stud*)))	229,779
30	((TI random* OR AB random*) N3 (TI allocat* OR AB allocat*))	6548
31	(TI placebo* OR AB placebo*)	38,396
32	(((TI singl* OR AB singl*) OR (TI doubl* OR AB doubl*) OR (TI trebl* OR AB trebl*) OR (TI tripl* OR AB tripl*)) W1 ((TI blind* OR AB blind*) OR (TI mask* OR AB mask*)))	29,489
33	((TI crossover* OR AB crossover*) OR ((TI cross OR AB cross) W1 (TI over* OR AB over*)))	14,410
34	(TI “‘field trial’“ OR AB “‘field trial’“)	1551
35	S28 OR S29 OR S30 OR S31 OR S32 OR S33 OR S34	258,642
36	S7 AND S12 AND S27 AND S35	136

### Directory of Open Access Journals

Date searched: January 14, 2023

**Table:**

Search	Search terms	Records retrieved
1	diabetes AND self-management AND trial AND Africa (all fields)	10

### OpenGrey

Date searched: August 6, 2021

**Table:**

Search	Search terms	Records retrieved
1	diabetes AND education AND trial	9

Date limit: inception–December 1, 2020.

### EThOS

Date searched: January 14, 2023

**Table:**

Search	Search terms	Records retrieved
1	diabetes AND education AND trial	41

### ProQuest Dissertations and Theses

Date searched: January 14, 2023

**Table:**

Search	Search terms	Records retrieved
1	ti(diabetes and self-management and trial)	4

### Cochrane CENTRAL

Date searched: January 14, 2023

**Table:**

Search	Search terms	Records retrieved
1	[mh “Diabetes Mellitus, Type 2”]	20,469
2	(type* NEAR/3 (‘2’ OR ‘II’ OR two*) NEAR/3 (diabete* OR diabetic*)):ti,ab	44,356
3	((late OR maturit* OR adult* OR slow*) NEAR/3 onset* NEAR/3 (diabete* OR diabetic*)):ti,ab	197
4	((‘ketosis-resistant*’ OR stable*) NEAR/3 (diabete* OR diabetic*)):ti,ab	501
5	(((“non” NEAR/2 insulin*) OR noninsulin*) NEAR/3 depend* NEAR/3 (diabete* OR diabetic*)):ti,ab	2254
6	(NIDDM OR T2DM OR T2D):ti,ab	12,821
7	#1 OR #2 OR #3 OR #4 OR #5 OR #6	49,904
8	((central OR eastern OR western OR southern) NEAR/1 africa*):ti,ab	310
9	((subsahara* OR (“sub” NEAR/2 sahara*)) NEAR/2 africa*):ti,ab	2195
10	(Angola OR Benin OR Botswana OR “‘Burkina Faso’“ OR Burundi OR “‘Cabo Verde’“ OR “‘Cape Verde’“ OR Cameroon OR “‘Central African Republic’“ OR Chad OR Comoros OR “‘Democratic Republic of Congo’“ OR “‘Republic of Congo’“ OR Congo OR “‘Cote Divoire’“ OR “‘Ivory Coast’“ OR “‘Equatorial Guinea’“ OR Eritrea OR Eswatini OR Swaziland OR Ethiopia OR Gabon OR Gambia OR Ghana OR Guinea OR ‘Guinea-Bissau’ OR Kenya OR Lesotho OR Liberia OR Madagascar OR Malawi OR Mali OR Mauritania OR Mauritius OR Mozambique OR Namibia OR Niger OR Nigeria OR Rwanda OR “‘Sao Tome’“ OR Senegal OR Seychelles OR “‘Sierra Leone’“ OR Somalia OR “‘South Africa’“ OR “‘South Sudan’“ OR Sudan OR Tanzania OR Togo OR Uganda OR Zambia OR Zimbabwe):ti,ab	18,658
11	#8 OR #9 OR #10	19,353
12	[mh “Life Style”]	6403
13	[mh “Health Education”]	21,440
14	[mh Exercise]	28,985
15	[mh Diet]	20,551
16	[mh “Nutrition Therapy”]	10,484
17	[mh ^“‘nutrition assessment’“]	0
18	[mh ^Food]	1352
19	[mh Meals] OR [mh “Dietary Carbohydrates”]	8163
20	(lifestyle* OR educat* OR knowledge OR exercise* OR fitness OR physical OR diet* OR nutrition OR food*):ti,ab	376,679
21	(‘non-pharmacolog*’ OR ‘nonpharmocolog’):ti,ab	4755
22	[mh “Patient Education as Topic”]	9302
23	[mh Self-Management]	741
24	[mh “Self Care”]	6181
25	(“‘self management’“ OR “‘self help’“ OR “‘self care’“ OR confidence OR “‘self efficacy’“ OR responsib* OR autonomy*):ti,ab	129,949
26	[mh Telemedicine]	3334
27	((tele NEAR/2 (health OR medicine OR care)) OR telehealth OR telemedicine OR telecare):ti,ab	4228
28	[mh “Cell Phone”]	2408
29	(“m-health” OR mhealth OR “‘mobile health’“ OR SMS OR messag* OR (“‘mobile” NEAR/2 phone*’)):ti,ab	18,748
30	[mh “Self-Help Groups”] OR [mh “Peer Group”] OR [mh “Social Support”]	5423
31	((peer OR patient OR emotional OR social OR psychosocial) NEAR/1 (support OR group*)):ti,ab	19,727
32	[mh “Medication Adherence”]	2790
33	(monitor* OR ‘self-monitor*’ OR selfmonitor*):ti,ab	101,215
34	((home OR environment* OR living OR assistive) NEAR/2 (adapt* OR modif* OR equipment OR technolog*)):ti,ab	1109
35	#12 OR #13 OR #14 #15 OR #16 OR #17 OR #18 OR #19 OR #20 OR #21 OR #22 OR #23 OR #24 OR #25 OR #26 OR #27 OR #28 OR #29 OR #30 OR #31 OR #32 OR #33 OR #34	563,540
36	#7 AND #11 AND #35	142
37	Trials only, not reviews	134

## Appendix II: Studies ineligible following full-text review, with reasons

### Reason for exclusion: Ineligible population (n=8)

1. Amendezo E, Timothy DW, Karamuka V, Robinson B, Kavabushi P, Nitrenganya C, *et al.* Effects of a lifestyle education program on glycemic control among patients with diabetes at Kigali University Hospital, Rwanda: a randomized controlled trial. Diabetes Res Clin Pract. 2017;126:129-37.
  *Reason for exclusion*: Mixed type 1 and type 2 diabetic population, and study authors note they are not able to distinguish between those groups.
2. Catley D, Puoane T, Tsolekile L, Resnicow K, Fleiming KK, Hurley EA, *et al.* Evaluation of an adapted version of the Diabetes Prevention Program for low- and middle-income countries: a cluster randomized trial to evaluate “Lifestyle Africa” in South Africa. PLoS Med. 2022;19(4):e1003964.
  *Reason for exclusion:* Prevention trial rather than people with preexisting type 2 diabetes.
3. Cooper H. Capturing the impact of patient education for people with type 2 diabetes [thesis]. University of Liverpool Department of Nursing; 2001.
  *Reason for exclusion*: Setting not explicitly stated but seems likely to be the UK.
4. Dyson P, *et al.* Dietary advice for people with diabetes: the role of carbohydrate in dietary treatment and an assessment of video education [thesis]. Oxford Brookes University; 2010.
  *Reason for exclusion*: UK setting.
5. Dunkley A. Metabolic syndrome and abdominal obesity: waist measurement and lifestyle education to reduce cardiovascular and diabetes risk [thesis]. University of Leicester; 2011.
  *Reason for exclusion*: UK setting.
6. Essien O, Otu A, Umoh V, Enang O, Hicks JP, Walley J. Intensive patient education improves glycaemic control in diabetes compared to conventional education: a randomised controlled trial in a Nigerian tertiary care hospital. PLoS One. 2017;12(1):e0168835.
  *Reason for exclusion*: Study authors unable to stratify by type 1 and type 2 diabetics.
7. Gregg, J. A randomized controlled effectiveness trial comparing patient education with and without acceptance and commitment therapy for type 2 diabetes self-management [thesis]. University of Nevada; 2004.
  *Reason for exclusion*: USA setting.
8. van Olmen J, Kegels G, Korachais C, de Man J, Van Acker K, Kalobu JC, *et al.* The effect of text message support on diabetes self-management in developing countries – a randomised trial. J Clin Translation Endocrinol. 2017;7:33-41.
  *Reason for exclusion*: Study authors unable to stratify by type 1 and type 2 diabetics.

### Reason for exclusion: Ineligible intervention (n=14)

1. Abutair A, Naser A, Hamed AT. The effect of soluble fiber supplementation on metabolic syndrome profile among newly diagnosed type 2 diabetes patients. Clin Nutr Res. 2018;7(1):31-9.
  *Reason for exclusion:* Assesses fiber supplementation father than a broader self-management intervention.
2. Ahmad A, Elnour AA, Yousif M, Farah FH. Pharmacist’s interventions to improve clinical outcomes in patients with type 2 diabetes mellitus: Nyala City, South Darfur State, Sudan. Int J Diabetes Dev Countries. 2015;35(4):578–87.
  *Reason for exclusion:* Pharmacist-based intervention that included optimization of pharmaceutical treatment as well as self-management support – unable to separate these effects.
3. Asuako B, Moses MO, Eghan BA, Sarpong PA. Fasting plasma glucose and lipid profiles of diabetic patients improve with aerobic exercise training. Ghana Med J. 2017;51(3):120-7.
  *Reason for exclusion:* Supervised exercise-intervention only, no self-management component.
4. Ezema C, Onwunali AA, Lamina S, Ezugwu UA, Amaeze AA, Nwankwon MJ, *et al.* Blood glucose response to aerobic exercise training program among patients with type 2 diabetes mellitus at the University of Nigeria Teaching Hospital, Enugu South-East, Nigeria. Int J Diabetes Dev Countries. 2015;35:88-94.
  *Reason for exclusion:* Supervised exercise-intervention only, no self-management component.
5. Ezema C, Omeh E, Onyeso OKK, Anyachukwu CC, Nwankwo MJ, Amaeze A, *et al.* The effect of an aerobic exercise programme on blood glucose level, cardiovascular parameters, peripheral oxygen saturation, and body mass index among southern Nigerians with type 2 diabetes mellitus, undergoing concurrent sulfonylurea and metformin treatment. Malays J Med Sci. 2019;26(5):88-97.
  *Reason for exclusion:* Supervised exercise-intervention only, no self-management component (diet education given to both intervention and control groups).
6. Fairall L, Folb N, Timmerman V, Lombard C, Steyn K, Bachmann MO, *et al.* Educational outreach with an integrated clinical tool for nurse-led non-communicable chronic disease management in primary care in South Africa: a pragmatic cluster randomised controlled trial. PLoS Med. 2016;13(11):e1002178.
  *Reason for exclusion:* The intervention applied is a training program and manual for nursing staff rather than a self-management intervention for people with type 2 diabetes.
7. Ikem R, Kolawole BA, Ojofeitimi EO, Salawu A, Ajose OA, Abiose S, *et al.* A controlled comparison of the effect of a high fiber diet on the glycaemic and lipid profile of Nigerian clinic patients with type 2 diabetes. Pak J Nutr. 2007;6(2):111-16.
  *Reason for exclusion:* Assesses the addition of extra fiber to the diet. There was an education component but this was present for both intervention and control groups, therefore does not assess a self-management intervention.
8. Maharaj S, Nuhu JM. Rebound exercise: a beneficial adjuvant for sedentary non-insulin-dependent type 2 diabetic individuals in a rural environment. Aust J Rural Health 2016;24(2):123-9.
  *Reason for exclusion:* Exercise-intervention only (supervised mini-trampoline exercises), no self-management component (diet education given to both intervention and control groups).
9. Mollentze W, Joubert G, Prins A, van der Linde S, Marx GM, Tsie KG. The safety and efficacy of a low-energy diet to induce weight loss, improve metabolic health, and induce diabetes remission in insulin-treated obese men with type 2 diabetes: a pilot RCT. Int J Diabetes Dev Countries. 2019;39(4):618-25.
  *Reason for exclusion:* Compares a low-energy diet to standard diet. There was an education component, but this was present in both arms, therefore does not assess a self-management intervention.
10. Mondal S, Gebeyehu M. Effects of aerobic and resistance exercises on selected physiological biochemical and anthropometric variables among type 2 diabetic patients in Dilla, Ethiopia. Indian J Public Health Res Dev. 2021;12(3):229.
  *Reason for exclusion:* Exercise-intervention only, no self-management component.
11. Myers B, Lombard CJ, Lund C, Joska JA, Levitt N, Naledi T, *et al.* Comparing dedicated and designated approaches to integrating task-shared psychological interventions into chronic disease care in South Africa: a three-arm, cluster randomised, multicentre, open-label trial. Lancet. 2022;400(10360):1321-33.
  *Reason for exclusion:* Psychological intervention for coping with stress and life problems, for reducing depression and alcohol use symptom severity.
12. Osho O, Akinbo SRA, Osinubi AA, Olawale OA. Effect of progressive aerobic and resistance exercises on the pulmonary functions of individuals with type 2 diabetes in Nigeria. Int J Endocrinol Metabol. 2012;10(1):411-17.
  *Reason for exclusion:* Supervised exercise-intervention only, no self-management component (education given to both intervention and control groups).
13. van Olmen J, Absetz P, Mayega RW, Timm L, Delobelle P, Alvesson HM, *et al.* Process evaluation of a pragmatic implementation trial to support self-management for the prevention and management of type 2 diabetes in Uganda, South Africa and Sweden in the SMART2D project. BMJ Open Diabetes Res Care. 2022;10(5):e002902.
  *Reason for exclusion:* Assesses comprehensive, broader interventions with some structural changes to care, beyond the scope of self-management.
14. Yan H, Prista A, Ranadive SM, Damasceno A, Caupers P, Kanaley JA, *et al.* Effect of aerobic training on glucose control and blood pressure in T2DDM East African males. ISRN Endocrinol. 2014:864897.

*Reason for exclusion:* Supervised exercise-intervention only, no self-management component.

### Reason for exclusion: Ineligible outcomes (n=9)

1. Adibe M, Aguwa CN, Ukwe CV. Cost-utility analysis of pharmaceutical care intervention vs usual care in management of Nigerian patients with type 2 diabetes. Value Health Reg Issues. 2013;2(2):189-98.
  *Reason for exclusion:* Does not report on any of the pre-specified primary outcome measures.
2. Erku D, Ayele AA, Mekuria AB, Belachew SA, Hailemeskel B, Tegegn HG. The impact of pharmacist-led medication therapy management on medication adherence in patients with type 2 diabetes mellitus: a randomized controlled study. Pharm Pract. 2017;15(3):1026.
  *Reason for exclusion:* Does not report on any of the pre-specified primary outcome measures.
3. Gopalan A, Paramanund J, Shaw PA, Patel D, Friedman J, Brophy C, *et al.* Randomised controlled trial of alternative messages to increase enrolment in a healthy food programme among individuals with diabetes. BMJ Open. 2016;6(11):e012009.
  *Reason for exclusion:* Does not report on any of the pre-specified primary outcome measures.
4. Hailu FB, Moen A, Hjortdahl P. Diabetes self-management education (DSME) - effect on knowledge, self-care behavior, and self-efficacy among type 2 diabetes patients in Ethiopia: a controlled clinical trial. Diabetes Metab Syndr Obes. 2019;12: 2489-99.
  *Reason for exclusion:* Does not report on any of the pre-specified primary outcome measures.
5. Muchiri J, Gericke G, Rheeder P. Impact of nutrition education on diabetes knowledge and attitudes of adults with type 2 diabetes living in a resource-limited setting in South Africa: a randomised controlled trial. J Endocrinol Metabol Diabetes S Afr. 2016;21(2):20-8.
  *Reason for exclusion:* Does not report on any of the pre-specified primary outcome measures.
6. Mutagwanya R, Nyago CM, Nakwagala FN. Effect of diabetes nutrition education on the dietary feeding practices and lifestyle of type 2 diabetic patients. Eur J Clin Nutr. 2022;76(2): 270-6.
  *Reason for exclusion:* Does not report on any of the pre-specified primary outcome measures.
7. Owolabi E, Ter Goon D, Ajayi AI. Efficacy, acceptability and feasibility of daily text-messaging in promoting glycaemic control and other clinical outcomes in a low-resource setting of South Africa: a randomised controlled trial. PLoS One 2019;14(11):e0224791.
  *Reason for exclusion:* Does not report on any of the pre-specified primary outcome measures.
8. Owolabi E, Ter Goon D, Ajayi AI. Impact of mobile phone text messaging intervention on adherence among patients with diabetes in a rural setting a randomized controlled trial. Medicine. 2020;99(12): e18953.
  *Reason for exclusion:* Does not report on any of the pre-specified primary outcome measures.
9. Umeifekwem J, Okwuosa C. Differential effects of two modes of exercise on anthropometric characteristics of persons with type-2 diabetes. J Biol Agricult Healthc. 2014;4(22): 67-74.

*Reason for exclusion:* Does not report on any of the pre-specified primary outcome measures.

### Reason for exclusion: Ineligible study design (n=8)

1. Adeniyi A, Uloko AE, Sanya AO, Fasanmade AA. Time course of improvement of metabolic parameters after a 12 week physical exercise programme in patients with type 2 diabetes: the influence of gender in a Nigerian population. BioMed Res Int. 2013;19:310574.
  *Reason for exclusion*: No control group.
2. Afemikhe, J, Chipps JA. An evaluation of a multidisciplinary patient centred type 2 diabetes self-management education programme in Edo state, Nigeria. Afr J Nurs Midwif. 2015;17:S165-S179.
  *Reason for exclusion*: Not randomized.
3. Assah F, Atanga EN, Enoru S, Sobngwi E, Mbanya JC. Community-based peer support significantly improves metabolic control in people with Type 2 diabetes in Yaounde, Cameroon. Diabet Med. 2015;32(7):886-9.
  *Reason for exclusion*: Not randomized.
4. Baumann L, Frederick N, Betty N, Josephine E, Agatha N. A demonstration of peer support for Ugandan adults with type 2 diabetes. Int J Behav Med. 2015;22(3):374-83.
  *Reason for exclusion*: No control group.
5. Abdelgadir M, Elbagir M, Eltom M, Berne C, *et al.* The influence of glucose self-monitoring on glycaemic control in patients with diabetes mellitus in Sudan. Diabetes Res Clin Pract. 2006;74(1):90-4.
  *Reason for exclusion*: Cross-sectional study, not randomized controlled trial.
6. Dahjio Y, Noubiap JJN, Azabji-Kenfack M, Essouma M, Loni GE, Onana AE, *et al.* Impact of a 12-week aerobic exercise training program on anthropometric and metabolic parameters of a group of type 2 diabetes Cameroonian women aged ≥50 years. Ann Transl Med. 2016;4(19):364.
  *Reason for exclusion*: No control group.
7. Fisher E, Boothroyd RI, Coufal MM, Baumann LC, Mbanya JC, Rotheram-Borus MJ, *et al.* Peer support for self-management of diabetes improved outcomes in international settings. Health Aff. 2012;31(1):130-9.
  *Reason for exclusion*: Evaluation of an intervention, not randomized controlled trial.
8. Kalweit K, Van Zyl DG, Rheeder P. Titrating insulin in patients with type 2 diabetes using a structured self-monitoring blood glucose regimen. S Afr Med J. 2018;108(8):654-9.

*Reason for exclusion*: No control group.

### Reason for exclusion: Study protocol (n=3)

1. Diriba D, Leung DYP, Suen LKP. A nurse-led, community-based self management program for people living with type 2 diabetes in Western Ethiopia: a feasibility and pilot study protocol. Diabet Med. 2021;38(8):e14587.
  *Reason for exclusion:* Potentially eligible full-text protocol; conference abstract available but no corresponding full text. Authors contacted but unable to provide data (undergoing peer review).
2. Lumu W, Kirbirige D, Wesonga R, Bahendeka S. Effect of nurse-led lifestyle choice and coaching intervention on systolic blood pressure among type 2 diabetes patients with a high atherosclerotic cardiovascular risk: study protocol for a cluster randomized trial. Trials. 2021;22(1):133.
  *Reason for exclusion:* Potentially eligible full-text protocol; no response to 2 attempts to contact authors by email.
3. Lygidakis C, Uwizihiwe JP, Kallestrup P, Bia M, Condo J, Vogele C. Community- and mHealth-based integrated management of diabetes in primary healthcare in Rwanda (D²Rwanda): the protocol of a mixed-methods study including a cluster randomised controlled trial. BMJ Open 2019;9:e028427.

*Reason for exclusion:* Potentially eligible full-text protocol; no response to 2 attempts to contact authors by email.

## Appendix III: Reports not retrieved, with reasons

Potentially eligible conference abstract; study authors contacted and unable to provide relevant full text (n=2). Potentially eligible conference abstract; full text could not be obtained; authors contacted 2 times with no response (n=3). Potentially eligible trial registration record; full text could not be obtained; authors contacted 2 times with no response (n=4). Potentially eligible trial registration record; authors contacted; no data available for inclusion in the review (n=2).

1. Ogbonna B. Can pharmaceutical care intervention improve lipid profile in type 2 diabetes patients; a prospective intervention study in a tertiary hospital in Nigeria. Value Health. 2016;19(7):A850. (Conference abstract).
2. Ogbonna B, Oparah A. Impact of pharmaceutical care interventions on type 2 diabetes in a tertiary hospital in Nigeria: exploring the frontier. Value Health 2016;19(3):A197. (Conference abstract).
3. Adisa R, Fakeye T. Pharmacists-based intervention strategies to address compliance problems among poorly controlled type 2 diabetes in ambulatory care settings in Nigeria. Int J Pharm Pract. 2012;20(Suppl1):41-2. (Conference abstract).
4. Lamptey R, Amoakoh-Coleman M, Klipstein-Grobusch K, Darko D, Agyepong IA, Acheampong F, *et al.* IDF21-0472 The effect of structured diabetes self-management education care on glycaemic control in Accra subsequent to COVID-19. Diabetes Res Clin Pract. 2022;186:109547. (Conference abstract).
5. Soin G, Kunyiha N, Shah J, Patel K, Arisi C, Njenga E, *et al.* A randomized trial using mobile short-text messaging to improve cardiovascular risk profile in poorly controlled diabetes in Kenya. Circulation. 2018;138(Suppl1). (Conference abstract).
6. Effect of motivational interviewing on glycemic control, self-care management and diabetes related distress among people with type 2 diabetes mellitus in Tigray region, Ethiopia: a randomized controlled trial. 2021. Registration number: PACTR202106626595700. (Trial registration).
7. Structured diabetes self-management education and care outcomes in adults living with type 2 diabetes in Accra, Ghana. 2021. Registration number: NCT04780425. (Trial registration).
8. The effect of nutrition education on nutritional status, body composition, health and health related quality of life of elderly population in Ilu Ababor Zone, South West Ethiopia. 2021. Registration number: PACTR202102840289918. (Trial registration).
9. Trial on an educative structured intervention by peer educators to improve HbA1c of patients with type 2 diabetes in the Sikasso region in Mali. 2010. Registration number: NCT01153048. (Trial registration).
10. Using mHealth (mobile health) to optimize glycemic control in adults with type 2 diabetes: proof of Concept Study. 2021. Registration number: NCT05013294. (Trial registration).
11. Mobile health intervention for improved adherence in type 2 diabetes. 2022. Registration number: NCT05291026. (Trial registration).

## Appendix IV: Characteristics of included studies

**Table:**

**Asante** ***et al.***,^27^ **2020**	
**Design**	Standard RCT (parallel design, individually randomized)
**Setting**	Ghana: outpatient clinic at Diabetes Centre of Komfo Anokye Teaching Hospital in Kumasi
**Participants**	Sample size: 60 Mean age in years (SD): I=55.1 (10.9); C=56.5 (9.8) Sex: 21.7% male Mean diabetes duration in years (SD): I=8.8 (6.8); C=8.2 (6.3) Existing diabetes treatment: participants were required to be on at least 1 oral antidiabetic drug without insulin. Data not provided on comorbidities/complications. Inclusion criteria: • age ≥18 • diagnosed with T2DM without any complication requiring immediate hospitalization • able to communicate in English • access to personal mobile phone and able to answer calls • HbA1c > 7% not more than 3 months before selection • mentally stable, with no vision, verbal, or hearing impairments • on oral hypoglycemic drugs without insulin Exclusion criteria: • other forms of diabetes (eg, type 1 or gestational diabetes) • insulin added to treatment during the study
**Intervention**	12-week intervention delivered by diabetes specialist nurse assisted by registered nurse, via telephone. Two calls per week for the first 4 weeks, then once weekly for a further 8 weeks. Mean duration of phone calls was 12 minutes. The calls were used to reinforce guidelines on diabetes self-management according to the book *Living with Diabetes* developed by Acheampong *et al.* in partnership with the University of Virginia and the Ministry of Health, Ghana. Call content included information on diet, exercise, medication taking, self-monitoring of blood glucose, foot care, and self-management goal evaluation. Each participant in the intervention group was allocated a diary where the interventionists recorded their call date, time, duration, personalized self-management goals, action plans, and self-management challenges. Remote, individual, delivered by professional
**Comparator**	Enhanced usual care Control group received usual care, plus all participants received a 1-day workshop to reinforce diabetes self-management education before the start of the intervention. All participants were advised to stick to their scheduled clinic appointments. Usual care included an outpatient specialist service with appointments every 1 to 6 months, depending on diabetes control and complications profile.
**Outcomes of relevance to the review questions**	HbA1c at 12 weeks
**Commercial funding**	No
**Primary categories of self-management intervention according to PRISMS taxonomy**	1) Education about condition and management 14) Lifestyle advice and support

**Table:**

**David** ***et al.***,^49^ **2021**	
**Design**	Standard RCT (parallel design, individually randomized)
**Setting**	Nigeria: outpatient diabetic clinic of Abubakar Tafawa Balewa University Teaching Hospital, Bauchi State Nigeria (tertiary health facility)
**Participants**	Sample size: 108 Mean age in years (SD): 50.1 (11.7) Sex: 31.5% male Median diabetes duration in years (IQR): 6 (3-9) Existing diabetes treatment: at least 1 antidiabetic drug (oral and/or insulin) Comorbidities and complications (n [%]): hypertension (80 [74.1]), dyslipidemia (73 [73.7]) Inclusion criteria: • clinically diagnosed T2DM patients with HbA1c ≥7% • at least 6 months regular clinic attendance prior to recruitment • age ≥18 • taking 1 or more antidiabetic medications for at least 6 months Exclusion criteria: • critically ill or unconscious • patients with blood disorders (lymphocytic leukemia, hemolytic anemia, hemoglobinopathy, chronic) • patients undergoing hemodialysis and on erythropoietin therapy or hematinic medications • pregnant women with diabetes mellitus • patients without mobile phone number
**Intervention**	Intervention group received 2 consecutive 30-45 minute face-to-face interviews and educational sessions at baseline and 3 months. Delivered by clinical pharmacist who was also a trained diabetes educator. Each participant in the intervention group was provided with diabetes-related information, including risk factors, complications, importance of healthy diet, physical activity, self-monitoring of blood glucose, adherence to prescribed medications, lifestyle modifications, and management of hypoglycemia. A copy of the educational package was given to each participant for reference. Participants were followed up via mobile phone calls/text messages every 6 weeks to review their previous session(s) and to be reminded of their clinic appointment date for data collection. Face-to-face, individual, delivered by professional
**Comparator**	Usual care Participants in the usual care group received care from physicians, nurses, and medication refill at the pharmacy department. They were interviewed by the clinical pharmacist and assessed at baseline but were not provided with the active intervention. Phone calls were made to remind them of their clinic appointment for data collection.
**Outcomes of relevance to the review questions**	HbA1c, FBG, SBP, DBP, total cholesterol, HDL, LDL, TG at 6 months
**Commercial funding**	No
**Primary categories of self-management intervention according to PRISMS taxonomy**	1) Education about condition and management 14) Lifestyle advice and support

**Table:**

**Debussche** ***et al.***,^18^ **2018**	
**Design**	Standard RCT (parallel design, individually randomized)
**Setting**	Mali, Bamako (capital city): consultation units located in secondary health centers (clinic-based)
**Participants**	Sample size: 151 Mean age in years (SD): 52.5 (9.8) Sex: 23.8% male Duration of diabetes not provided. Existing diabetes treatment: included patients with no treatment/diet only, oral antidiabetic drugs, and those on insulin. Data not provided on comorbidities/complications. Inclusion criteria: • aged between 30 and 80 years • underwent regular follow-ups and monitoring in Bamako consultation units for poorly controlled T2DM (HbA1c 8%) • afreed to attend all peer-led educational sessions • agreed to have their clinical and biological measurements taken until completion of the protocol Exclusion criteria: • T1DM • severe complications in the 3 months preceding enrollment (infections, severe renal failure, coronary events, foot lesions) • concomitant illnesses that threatened functional or vital prognosis
**Intervention**	Three courses delivered in the community by trained peer educators over 1 year to groups of 4-10. Each course was offered over a period of 3 months and covered 4 different thematic sessions. Session durations were 1.5-2 hours using an empowerment-based approach. Themes addressed included cardiovascular risk management, food-intake, exercise, blood glucose, and insulin management. The content, approach, and program of each group session were detailed in specific booklets for learners (including learners with literacy difficulties) and culturally adapted for Mali (food habits, language specificities, occupational and environment issues). Peer educators were recruited from the local association of diabetic patients. Initial 4-day training program with assessments. The best performing 5 peer educators out of the group of 10 were selected to deliver the intervention. Face-to-face, group-based, delivered by peer educators
**Comparator**	Usual care Control group received conventional care alone, which included individual counseling sessions, measurement of blood glucose, weight and blood pressure, data collection, clinical examination, and prescription or renewal of treatment.
**Outcomes of relevance to the review questions**	HbA1c, weight, BMI, SBP and DBP at 6 and 12 months; WC at 12 months
**Commercial funding**	No
**Primary categories of self-management intervention according to PRISMS taxonomy**	1) Education about condition and management 13) Social support 14) Lifestyle advice and support

**Table:**

**Farmer** ***et al.***,^28^ **2021**	
**Design**	Standard RCT (parallel design, individually randomized)
**Setting**	South Africa and Malawi Cape Town: large primary care clinic serving 2 low-income communities to the north of the city Lilongwe: hospital-based outpatient clinic
**Participants**	Sample size: 1186 Mean age in years (SD): 57.1 (11.4) Sex: 30.1% male Median duration of diabetes in years (IQR): 5.0 (2.8, 10.0) Existing diabetes treatment: patients were required to be taking an oral glucose-lowering medication. Some participants on insulin only were inadvertently recruited then excluded from the analysis. Comorbidities and complications (n [%]): heart attack (64 [5.7]), heart failure (48 [4.3]), TIA (86 [7.7]), angina/chest pain (91 [8.1]), peripheral vascular disease (144 [12.9]), CKD (39 [3.5]), TB (105 [9.4]), mental illness (54 [4.8]) Inclusion criteria: • T2DM • age ≥18 years • taking an oral glucose-lowering medication • able to communicate in 1 of the predominant official languages spoken in the Western Cape province in South Africa (English, Afrikaans, or IsiXhosa) and Malawi (English or Chichewa) • access to a mobile phone (shared access permitted with permission of phone owner) • able to send and receive text messages (or be helped to) • current and planned future residence in participating clinic communities Exclusion criteria: • admission with hyperglycemia or hypoglycemia in the last 3 months • pregnant or within 3 months postpartum, or plans to become pregnant in next 12 months • terminal medical condition • household member of someone already recruited to the trial • participated in formative work for the trial
**Intervention**	The intervention was automated and consisted of motivational and educational text messages sent to participants on different days, 3-4 times weekly over a period of 12 months. Message content was intended to encourage people to take their medicine regularly as prescribed (70% of the messages), alongside other information intended to provide advice about healthy lifestyle and enhancing well-being (30% of the messages). Specific messages encouraged people to check the date of their next appointment and whether they had sufficient medication. Message content was developed from lived experience of diabetes, diabetes treatment services, and expert opinion and formulated as SMS text messages. Remote, individual, automated text messaging
**Comparator**	Sham intervention Control group received usual care supplemented by “active control.” Participants allocated to the usual care group were sent a research-related message thanking them for taking part in the study and providing trial-related information every 6 weeks. Routine clinical care consisted of attendance to collect medication supplies at regular intervals. Health material on T2DM was available at all sites and included information about the importance of taking medicine regularly, alongside other health information.
**Outcomes of relevance to the review questions**	HbA1c, HRQoL, adverse events, BMI, SBP and DBP at 12 months
**Commercial funding**	No
**Primary categories of self-management intervention according to PRISMS taxonomy**	6) Practical support with adherence (medication or behavioral)

**Table:**

**Fayehun** ***et al.***,^20^ **2018**	
**Design**	Standard RCT (parallel design, individually randomized)
**Setting**	Nigeria: general outpatient clinic at University College Hospital, Ibadan
**Participants**	Sample size: 46 Mean age in years (SD): 54.0 (7.7) Sex: 37% male Duration of diabetes: <7 years 69.6%; ≥7 years 30.4% Existing diabetes treatment: dietary control with or without oral hypoglycemic agents Data not provided on comorbidities/complications Inclusion criteria: • age 18-64 years • T2DM for at least 12 months • non-insulin dependent • could walk without limitations or pain Exclusion criteria: • pregnant women • smokers • individuals on prescription medication that might impair ability to walk
**Intervention**	The intervention group participants were given the goal of accumulating 10,000 steps per day during the 10-week intervention period. They were counseled to increase their daily step count by 20% from baseline each week, until the 10,000 steps goal was reached. Possible motivators and barriers to walking were identified. Additional counseling was given at the 4- and 8-week visit, and telephone follow-up at weeks 2, 6, and 10. Face-to-face, unclear if group or individual, unclear if delivered by professional/lay person.
**Comparator**	Enhanced usual care. Control group participants were asked to maintain their normal activity habits and encouraged to keep a daily step count during follow-up.
**Outcomes of relevance to the review questions**	HbA1c at 10 weeks
**Commercial funding**	No
**Primary categories of self-management intervention according to PRISMS taxonomy**	12) Training/rehearsal for psychological strategies 14) Lifestyle advice and support

**Table:**

**Gathu** ***et al.***,^21^ **2018**	
**Design**	Standard RCT (parallel design, individually randomized)
**Setting**	Kenya: family medicine clinic at Aga Khan University Hospital, Nairobi
**Participants**	Sample size: 140 Mean age in years (SD): 48.8 (9.8) Sex: 56% male Duration of diabetes: <5 years 49.3%; 5-10 years 22.1%; >10 years 28.6% Existing diabetes treatment: included patients treated with diet only, oral antidiabetic drugs, and those on insulin. Comorbidities and complications (n [%]): hypertension (54 [39]). Additional data not provided (those with complications were excluded). Inclusion criteria: • sub-optimally controlled T2DM defined as HbA1c ≥8% • age 18–65 years Exclusion criteria: • other types of diabetes that were not T2DM • diabetes-related complications • anemia at last Hb check
**Intervention**	The intervention group received referral to a certified diabetes educator for individualized structured DSME training. Two certified diabetes educators with level 4 designation offered the individualized DSME sessions. The education content included the American Association of Diabetes Educators 7-core self-care behaviors, involving 3 1-hour sessions every 6 weeks. At the end of the sessions, participants received a patient guide to diabetes booklet and graphic material illustrating several self-care activities, such as foot care. Subsequent consultations were mainly feedback sessions aimed at reviewing previously discussed matters, reinforcing key messages, addressing challenges, and providing additional information. Participants also received telephone reminders a week prior to their scheduled appointment with the diabetes educators. A hotline number was provided to consult with the diabetic educator at any given time of the day. Face-to-face and remote, individual, delivered by professional
**Comparator**	Usual care Control group received usual care with no modification. This was delivered by the family physician and consisted of a 20–30-minute standard doctor consultation where the recent HbA1c level and medication compliance were reviewed, and a brief informal patient-tailored diabetes education was offered.
**Outcomes of relevance to the review questions**	HbA1c at 10 weeks HbA1c, BMI, SBP, and DBP at 6 months
**Commercial funding**	Not specified
**Primary categories of self-management intervention according to PRISMS taxonomy**	1) Education about condition and management 14) Lifestyle advice and support

**Table:**

**Hailu** ***et al.***,^50^ **2018 and Hailu** ***et al.***,^51^ **2021**	
**Design**	Standard RCT (parallel design, individually randomized)
**Setting**	Ethiopia: Jimma University Medical Centre (JUMC). Participants were recruited from Jimma city and rural districts surrounding Jimma city in Southwest Ethiopia.
**Participants**	Sample size: 220 Mean age in years (SD): I=55 (10); C=55 (14) Sex: 67.2% male Mean diabetes duration in years (SD): I=10 (6); C=12 (7) Existing diabetes treatment: all participants on either oral antidiabetic drugs, insulin, or both. Data not provided on comorbidities/complications. Inclusion criteria: • age >30 years at time of T2DM diagnosis • overweight or obese • taking oral hypoglycemic drugs with or without insulin Exclusion criteria: • T1DM or GDM • pregnant women • severe cognitive or physical impairment • terminally ill
**Intervention**	Entailed 6 DSME sessions for approximately 1.5 hours every month for 6 consecutive months. Delivered by a PhD nurse student and 1 clinical nurse fluent in the local languages. They had been trained for a total of 16 hours. The training was supported by handbooks and fliers with colorful, illustrative pictures customized to the local context and patients’ literacy level. Nurses facilitated brief education on the specific session topic, led discussion, facilitated experience sharing among participants, concluded the session, gave take-home activities, and reviewed how the participants were undertaking take-home activities. Face-to-face, group-based, delivered by professional.
**Comparator**	Usual care The control group received usual care (not detailed).
**Outcomes of relevance to the review questions**	HbA1c and HRQoL at 9-11 months
**Commercial funding**	No
**Primary categories of self-management intervention according to PRISMS taxonomy**	1) Education about condition and management 14) Lifestyle advice and support

**Table:**

**Mash** ***et al.***,^22^ **2014**	
**Design**	Cluster RCT
**Setting**	South Africa: participants were recruited from 45 community health centers in the Cape Town Metropole.
**Participants**	Sample size: 1570 (34 clusters) Mean age in years (SD): 56.1 (11.6) Sex: 26.2% male Diabetes duration: not provided Existing diabetes treatment: participants were on oral antidiabetic drugs and/or insulin. Comorbidities and complications (n [%]): hypertension I=539 (75.9), C=715 (83.1); hypercholesterolemia I=239 (33.7), C=279 (32.4); CKD I=12 (1.7), C=26 (3.0); cataracts I= 67 (9.4), C=83 (9.7); retinopathy I=40 (5.6), C=5 (0.6); peripheral vascular disease I=13 (1.8), C=1 (0.1); leg ulcers I=24 (3.4), C=36 (4.2); neuropathy I=52 (7.3), C=12 (1.4); amputation I=8 (1.1), C=7 (0.8); ischemic heart disease I=22 (3.1), C=26 (3.0); cardiac failure I=4 (0.6), C=35 (4.1); stroke I=20 (2.8), C=26 (3.0) Inclusion criteria: • patients with T2DM attending the selected health center on recruitment days Exclusion criteria: • patients with T1DM • refused consent • judged unable to participate (eg, those who were acutely ill)
**Intervention**	A total of 4 × 60-minute monthly sessions of group diabetes education led by a health promoter focused on understanding diabetes, living a healthy lifestyle, understanding medication, and avoiding complications. Health promoters were “mid-level health workers,” recruited from the district health services and trained over a total of 6 days. Written resource materials also provided. Face-to-face, group-based, delivered by professional.
**Comparator**	Usual care Patients in the control group received usual education at the health center. Usual education consisted of ad hoc educational talks in the waiting or club room as well as any individual counseling that providers might have time for in the consultation.
**Outcomes of relevance to the review questions**	HbA1c, HRQoL, weight, WC, SBP, DBP, and total cholesterol at 12 months
**Commercial funding**	No
**Primary categories of self-management intervention according to PRISMS taxonomy**	1) Education about condition and management 14) Lifestyle advice and support

**Table:**

**Muchiri** ***et al.***,^23^ **2016**	
**Design**	Standard RCT (parallel design, Individually randomized)
**Setting**	South Africa: participants were recruited from 2 community health centers in Moretele sub-district, North-West Province.
**Participants**	Sample size: 82 Mean age in years (SD): 58.8 (7.7) Sex: 15.5% male Median diabetes duration in years (IQR): I=5 (3-9); C=7 (4-10) Existing diabetes treatment: included patients on oral antidiabetic drugs; those on insulin were excluded. Data not provided on comorbidities/complications. Inclusion criteria: • at least 1 year living with diabetes • blood sugar >10 mmol/L or above on 2 occasions in previous 6 months • regular attendance at diabetic clinic • HbA1c ≥ 8% Exclusion criteria: • on insulin therapy • pregnant • in full-time employment • planning to move from the study area during study period
**Intervention**	A dietician-led nutritional education program undertaken in groups of 6-10, consisting of 3 components: i) education on the curriculum (8 weekly sessions, 2 to 2.5 hours each) ii) follow-up sessions (4 monthly meetings and 2 bi-monthly meetings each lasting 1.5 hours) iii) vegetable gardening (demonstration of sowing/transplantation of vegetables) Face-to-face, group-based, delivered by professional.
**Comparator**	Enhanced usual care The control group participants received education materials that were also given to the intervention group (pamphlet and wall/fridge poster) and continued with usual medical care at their community health center.
**Outcomes of relevance to the review questions**	HbA1c, BMI, SBP, DBP, total cholesterol, HDL, LDL at 6 and 12 months
**Commercial funding**	Yes South African Sugar Association (grant number 212) Nestlé Nutrition Institute Africa
**Primary categories of self-management intervention according to PRISMS taxonomy**	1) Education about condition and management 14) Lifestyle advice and support

**Table:**

**Muchiri** ***et al.***,^48^ **2021**	
**Design**	Standard RCT (parallel design, individually randomized)
**Setting**	South Africa: participants were recruited from a diabetes outpatient clinic at a public tertiary teaching hospital located in Pretoria.
**Participants**	Sample size: 77 Mean age in years (SD): 57.2 (6.6) Sex: 22.1% male Median diabetes duration in years (IQR): I=12 (6–20); C=17 (9–23) Existing diabetes treatment: included patients on oral antidiabetic drugs, insulin, and both combined Comorbidities and complications (n [%]): hypertension: I=38 (97.4), C=36 (94.7); heart diseases: I=12 (30.8), C=11 (29.0); dyslipidemia: I=24 (61.5), C=22 (57.9); nephropathy: I=5 (12.8), C=4 (10.5); retinopathy: I=2 (5.1), C=2 (5.3) Inclusion criteria: • age 40-70 years • HbA1c ≥8% • at least 1 year of living with diabetes • able to understand English Exclusion criteria: • pregnant • employed full-time • planning to be out of the study site in the next year • major complications (proliferative retinopathy, severe renal insufficiency [eGFR<15], amputations)
**Intervention**	The intervention group received a nutrition education program delivered by a dietician and assisted by a nutrition MSc student, delivered over 1 year. The program comprised 4 components: i) 7 monthly group training sessions based on a diet-focused curriculum and supported by a training manual ii) 1 individual counseling and goal-setting session iii) Group follow-up sessions (bi-monthly) iv) A workbook with a summary of key messages and activities to engage participants between sessions Group sizes were 4-7; durations of sessions not specified. Face-to-face, group-based and individual, delivered by professional.
**Comparator**	Enhanced usual care Both the intervention and control groups received education materials comprising a pamphlet and a wall/fridge poster. Both groups continued with usual medical care at the diabetes outpatient clinic. Participants in the control group had no further encounters except for outcome assessments.
**Outcomes of relevance to the review questions**	HbA1c, BMI, SBP, DBP, total cholesterol, HDL, LDL, TG at 6 and 12 months
**Commercial funding**	Yes: South African Sugar Association (grant number 251)
**Primary categories of self-management intervention according to PRISMS taxonomy**	1) Education about condition and management 14) Lifestyle advice and support

**Table:**

**Ng’ang’a** ***et al.***,^52^ **2022**	
**Design**	Standard RCT (parallel design, individually randomized).
**Setting**	Rwanda: 3 public district-level hospitals in rural Rwanda: Kirehe and Rwinkwavu district hospitals in the Eastern Province, and Butaro district hospital in the Northern Province.
**Participants**	Sample size: 80 Median age in years (IQR): I=53.2 (41.3-58.3); C=50.4 (39.4-62.4) Sex: 43.8% male Median diabetes duration in years (IQR): I=9 (3–14); C=9 (6–12) Existing diabetes treatment: included patients on insulin with or without oral antidiabetic drugs Data not provided on comorbidities/complications Inclusion criteria: • age ≥18 years • diagnosed with insulin-dependent T2DM • receiving an insulin regimen at the time of the study at 1 of the 3 district hospitals which formed the study sites Exclusion criteria: • T1DM • GDM • CKD • unable to read and write sufficiently to use logbooks and had no reliable person to assist them
**Intervention**	SMBG intervention. Participants in the intervention group were given SMBG kit and received training targeted towards SMBG, including appropriate use of the kit, proper waste-disposal mechanisms, signs and symptoms of hypo- and hyperglycemia, and how to manage these. Participants were also given a mobile phone number belonging to the study coordinator to communicate in case of any concerns or questions related to implementing the SMBG. Face-to-face and remote, unclear if group or individual, unclear if delivered by professional/lay person.
**Comparator**	Usual care Both the intervention and control groups continued with their usual diabetes management, which consisted of routine monthly medical consultation and education. The control group did not receive any additional changes to its day-to-day care.
**Outcomes of relevance to the review questions**	HbA1c at 6 months
**Commercial funding**	No
**Primary categories of self-management intervention according to PRISMS taxonomy**	5) Monitoring of condition with feedback to the patient 11) Training/rehearsal for practical self-management activities

**Table:**

**Ojieabu** ***et al.***,^17^ **2017**	
**Design**	Standard RCT (parallel design, individually randomized)
**Setting**	Nigeria: endocrinology clinic of Olabisi Onabanjo University Teaching Hospital (OOUTH), Sagamu, Ogun State, Nigeria (government-owned tertiary hospital)
**Participants**	Sample size: 150 Age (n [%]): 50‑59 years: I=21 (28.0), C=17 (22.7); 60‑69 years: I=38 (50.7), C=41 (54.6); >69 years 17: I=16 (21.3), C=17 (22.7) Sex: 38% male Diabetes duration: not provided Existing diabetes treatment: not provided Data not provided on comorbidities/complications Inclusion criteria: • T2DM • on hypoglycemic medication for more than 3 months • receiving medical care from OOUTH for diabetes • age ≥50 years Exclusion criteria: • mental incompetence • acute illness • comorbidities other than hypertension • declined participation
**Intervention**	Pharmacist education program. Phone calls a week before and a day before their clinic visit day. At least 4 sessions of education with the pharmacist over a 4-month period. This included information on diabetes and hypertension, their complications, risks, preventive measures and management, the need for medication and treatment adherence such as clinic visits, and lifestyle modifications including diet and exercise. Durations of sessions not specified. Face-to-face, unclear if group-based or individual, delivered by professional
**Comparator**	Usual care All patients received the usual general briefing of about 10-15 minutes from a coordinating staff member on each clinic day. The control group was deprived of the pharmacist‑led education and counseling sessions throughout the period of the study.
**Outcomes of relevance to the review questions**	FBG, BMI, SBP, and DBP at 4 months
**Commercial funding**	Not specified
**Primary categories of self-management intervention according to PRISMS taxonomy**	1) Education about condition and management 6) Practical support with adherence (medication or behavioral)

**Table:**

**Ojieabu,** ^53^ **2020**	
**Design**	Standard RCT (parallel design, individually randomized)
**Setting**	Nigeria: endocrinology clinic of Olabisi Onabanjo University Teaching Hospital (OOUTH), Sagamu, Ogun State, Nigeria (government-owned tertiary hospital)
**Participants**	Sample size: 170 Age (n [%]): 50–69 years: I=57 (67.1), C=52 (61.2); >69 years: I=28 (32.9), C=33 (38.8) Sex: 39.4% male Diabetes duration: not provided Existing diabetes treatment: not provided Data not provided on comorbidities/complications Inclusion criteria: • T2DM • on hypoglycemic medication for more than 3 months • receiving medical care from OOUTH for diabetes • age ≥50 years Exclusion criteria: • mental incompetence • acute illness • comorbidities other than hypertension • declined participation
**Intervention**	Pharmacist-led patient education/counseling. Topics included: diabetes and hypertension prevention and risk factors, hyperglycemia and hypoglycemia, including pharmacological and non-pharmacological management. Patients were also counseled on the advantages of medication adherence and clinic attendance as well as lifestyle modifications. Brisk walking was demonstrated at least 4 times during the study period. At least 4 sessions were held with each patient. Durations of sessions not specified. Face-to-face, unclear if group-based or individual, delivered by professional.
**Comparator**	Usual care Control group was deprived of intervention strategies, apart from usual phone call reminders for appointment days.
**Outcomes of relevance to the review questions**	HRQoL at 4 and 8 months
**Commercial funding**	No
**Primary categories of self-management intervention according to PRISMS taxonomy**	1) Education about condition and management 6) Practical support with adherence (medication or behavioral)

**Table:**

**Pienaar** ***et al.***,^29^ **2021**	
**Design**	Cluster RCT
**Setting**	South Africa: 6 communities in a semi-urban rural area in Free State
**Participants**	Sample size: 288 (6 community clusters) Median age in years (IQR): I=60 (54-69), C=62 (56-69) Sex: 16.0% male Median diabetes duration in years (IQR): I= 6 (3–12); C= 8 (3–14) Existing diabetes treatment: not provided Comorbidities and complications (n[%]): cardiovascular: I = 115 (95.8), C = 129 (97.7); asthma: I = 2 (1.7), C = 5 (3.8); epilepsy: I = 2 (1.7), C = 2 (1.5); mental illness: I = 4 (3.3), C = 0; HIV/AIDS: I = 4 (3.3), C = 3 (2.3); arthritis: I = 6 (5.0), C = 5 (3.8); gastrointestinal: I = 0 (0), C = 1 (0.8); renal: I = 1 (0.8), C = 0 (0) Inclusion criteria: • age ≥18 years • diagnosed with T2DM by a physician • home language: Sesotho Exclusion criteria: • acutely ill • known psychiatric/psychological disorders that may impair judgment and memory
**Intervention**	Peer-support from trained CHWs for 4 months. CHW training included monthly interactive training sessions lasting 60-120 minutes for 4 months at the respective PHCs. The CHWs facilitated monthly face-to-face group sessions lasting about 60 minutes at PHCs. Group size approximately 5. The CHWs also conducted home visits once a month to reinforce knowledge and skills, to listen to the concerns of the individuals, and to work with them to solve problems. CHWs worked in pairs to support each other during group and home visits. Face-to-face, group-based, and individual, delivered by lay person (CHWs = peer educators)
**Comparator**	Usual care Usual care involved collecting medication at the PHC every month, random health talks in the waiting area, and consultation with the clinical nurse practitioner or doctor once every 3 months.
**Outcomes of relevance to the review questions**	HbA1c, BMI, WC, SBP, and DBP at 4 months
**Commercial funding**	No
**Primary categories of self-management intervention according to PRISMS taxonomy**	1) Education about condition and management 13) Social support 14) Lifestyle advice and support

**Table:**

**Thuita** ***et al.***,^24^ **2020**	
**Design**	Standard RCT (parallel design, individually randomized, 3 arms)
**Setting**	Kenya: diabetes comprehensive care center (DCC) at Thika Level 5 Hospital in Kiambu Country
**Participants**	Sample size: 153 Mean age in years (SD): NEP group = 57.0 (10.9); NE group = 55.0 (12.3); C = 56.0 (12.0) Sex: 40.5% male Mean diabetes duration in years (SD): NEP group = 6.0 (7.1); NE group = 7.0 (6.9); C = 7.0 (6.0) Existing diabetes treatment: included patients on oral antidiabetic drugs, insulin, and both combined. Comorbidities and complications (n [%]): foot disease: NEP = 5 (9.8), NE = 7 (13.7), C = 5 (9.8); eye problems: NEP = 13 (25.5), NE = 12 (23.5), C = 11 (21.6); kidney problems: NEP = 0 (0), NE = 2 (3.9), C=0 (0); neuropathy: NEP = 1 (2.0), NE = 0 (0), C = 3 (5.9); arthritis: NEP = 6 (11.8), NE = 7 (13.7), C = 5 (9.8) Inclusion criteria: • age 20-79 years • T2DM • regular attendance at the DCC Exclusion criteria: • pregnant • planning to move from study area during the study period • complications such as renal failure, congestive cardiac failure, or stroke
**Intervention**	Intervention groups (NE and NEP) underwent a nutrition education program for 8 weeks, which also covered the importance of physical activity (NE group). In addition, the NEP group was trained on peer-to-peer support. The nutrition education given to the NE and NEP intervention groups included weekly (120 minutes each) nutrition classes conducted over 8 weeks by the PI. The nutrition education curriculum was developed by the PI after review of related literature on nutrition management of T2DM. The PI was a nutritionist. Participants in the NEP group were divided into small support groups (5-10 participants), depending on the location they came from as well as their age. After each education session, members of the support groups were encouraged to share other weekly goals for specific changes in their eating and physical activity behavior with one another. After the 8-week training, participants were followed monthly, and they presented their progress and new goals to the group members for a period of 6 months. A trained peer educator living with diabetes for 13 years from Kenya Defeat Diabetes Association joined the PI during the monthly meetings and encouraged the participants in the peer-support groups by sharing his experiences. Face-to-face, group-based, delivered by professional and lay person.
**Comparator**	Enhanced usual care All study participants received standard education that covered content on diabetes pathophysiology, risk factors, symptoms, complications, hyperglycemia and hypoglycemia symptoms, and foot care treatment goals and modalities. This was done by the PI together with a clinician who runs the clinic.
**Outcomes of relevance to the review questions**	HbA1c, FBG, weight, BMI, WC, SBP, DBP, total cholesterol, HDL, LDL and TG at 6 months
**Commercial funding**	No
**Primary categories of self-management intervention according to PRISMS taxonomy**	1) Education about condition and management 13) Social support 14) Lifestyle advice and support

**Table:**

**van Rooijen** ***et al.***,^54^ **2004**	
**Design**	Standard RCT (parallel design, individually randomized)
**Setting**	South Africa: clinic-based; Mamelodi Community Hospital, East of Pretoria, Guateng Province
**Participants**	Sample size: 158 Mean age in years: I=55, C=54, SDs not provided Sex: 0% male Diabetes duration: not provided Existing diabetes treatment: included patients with no treatment/diet only, oral antidiabetic drugs, and those on insulin Comorbidities and complications (n [%]): hypertension: I = 53 (66.3), C = 45 (58.4); arthritis: I = 1 (1.3), C = 2 (2.6); combination (unspecified): I = 8 (10), C = 9 (11.7) Inclusion criteria: • women only • age 40-65 years • T2DM • duration of diabetes of at least 1 year Exclusion criteria: • nil additional
**Intervention**	The intervention consisted of an incremental daily home exercise program; the use of daily physical activity records, and 6 fortnightly supervised aerobic exercise classes. Participants were encouraged to form small groups of women living near each other to join in the exercises. Patients were instructed to increase walking at home from 10- to 45-minute sessions over the 12 weeks of training. Subjects were instructed to walk twice a day, starting with 5 minutes per session, and to increase their total daily walking time by 10 minutes every 2 weeks, up to 45 minutes per day. Subjects in the exercise group were instructed to keep a daily record of the time they spent on each of the activities in the diary. Education component within the exercise program: the fortnightly exercise sessions of 45 minutes each at the Mamelodi Hospital were used to educate the subjects about exercise, to demonstrate the home exercises, and to address problems experienced with home programs. Face-to-face and remote, group-based and individual, unclear if delivered by professional/lay person.
**Comparator**	Sham intervention Relaxation-based: the education was the same for both the exercise and the relaxation groups and consisted of interactive group sessions on the same day as the intervention at the hospital. A registered dietician gave lectures on food portion size and use of fat, fiber, and salt in the diet. The relaxation group was also required to visit the hospital fortnightly, as did the exercise group. The subjects in the relaxation group did not receive any home exercises and were not advised to exercise at home. Subjects were instructed to progressively tense, and then relax alternating muscle groups. The duration of the relaxation exercises was 20 minutes per session.
**Outcomes of relevance to the review questions**	HbA1c, adverse events, BMI, SBP, and DBP at 12 weeks
**Commercial funding**	Yes: partially sponsored by the South African Sugar Association
**Primary categories of self-management intervention according to PRISMS taxonomy**	11) Training/rehearsal for practical self-management activities 14) Lifestyle advice and support

**Table:**

**van Rooijen** ***et al.***,^55^ **2010**	
**Design**	Standard RCT (parallel design, individually randomized)
**Setting**	South Africa: diabetes outpatient clinic of the Steve Biko Academic Hospital in Gauteng Province (tertiary level clinic)
**Participants**	Sample size: 51 Mean age in years (SD): I = 53.2 (6.4), C = 54.1 (6.3) Sex: 41.2% male Diabetes duration in years (n [%]): 1-4 years: I = 18.5 (5), C = 8.3 (2); 5-9 years: I = 37 (10), C = 12.5 (3); >10 years: I = 44.4 (12), C = 79.2 (19) Existing diabetes treatment: included patients with no treatment/diet only, oral antidiabetic drugs, and those on insulin. Data not provided on comorbidities/complications. Inclusion criteria: • age 40-65 years • T2DM for at least 1 year • HbA1c 8-9.5% inclusive Exclusion criteria: • unable to read
**Intervention**	Participants attended 4 weekly group sessions. A staged approach to education based on the Skilled-Helper Model was used to empower the participants with knowledge and skills necessary for medical nutrition therapy, with the ultimate goal to help participants to become better at helping themselves in their everyday lives. Topics included planning, purchasing, and preparing food and meals; sources of carbohydrates, protein, and fat; reading of food nutrition labels; grocery shopping guidelines; modifying fat intake; use of sugar-containing foods; diabetic foods and sweeteners, as well as the glycemic index and glycemic load. No individualized dietary programs were given. Each subject in this group received a Yamax SW-200 pedometer. Participants had to wear the sealed pedometers from the time they woke up until they went to bed at night for 2 weekdays and 1 weekend day to establish the baseline average number of steps for each patient. Personal goal setting was calculated on the average of the 3-day step record. After the 4 group sessions, participants continued at home from week 5 of the program. They received motivating text messages fortnightly. Participants were also encouraged to phone or visit the researchers at the clinic whenever they had problems or questions. The participants had a follow-up assessment at 16 weeks, during which time they had to return the pedometers, but continued with their respective walking and eating programs. Final assessment was conducted at 1 year. Face-to-face and remote, group-based, unclear if delivered by professional or lay person.
**Comparator**	Usual care The control group received usual care and were waitlisted for an intervention similar to the intervention group after the study was completed.
**Outcomes of relevance to the review questions**	HbA1c, adverse events, BMI, total cholesterol, HDL, LDL, and TG at 16 weeks and 1 year
**Commercial funding**	Yes: partially sponsored by the South African Sugar Association
**Primary categories of self-management intervention according to PRISMS taxonomy**	14) Lifestyle advice and support

**Table:**

**Wargny** ***et al.***,^56^ **2018**	
**Design**	Cluster crossover design
**Setting**	Senegal: 2 medical centers: the Centre Philippe Maguilène Senghor (S) located in Yoff, in the northwestern suburbs of Dakar, and the Centre Médical de Popenguine (P), 70 km south of Dakar
**Participants**	Sample size: 191 (2 clusters) Mean age in years (SD): S=55 (10), *P*=54 (12) Sex: 20.4% male Diabetes duration in years (n [%]): <1: S = 19 (21), P = 15(16); 1–5: S = 28 (30), P = 42 (46); 5–10: S = 24 (26), P = 19 (21); >10: S = 21 (23), P = 16 (17) Existing diabetes treatment: inclusive of those treated with insulin; data on oral hypoglycemics/diet control not provided Data not provided on comorbidities/complications. Inclusion criteria: • age ≥18 years • T2DM • mobile able to receive SMS Exclusion criteria: • sickle cell disease
**Intervention**	SMS-based intervention. The S center participants received SMS during the first 3 months (M0 to M3) then no SMS during the 3 following months (M3 to M6), while it was the reverse for P center participants (no SMS from M0 to M3, then SMS from M3 to M6). The same SMS series (50 SMS overall) were sent to participants in each center, with a daily SMS on weeks 1, 2, 4, 5, 7, 9, and 11, and no SMS in weeks 3, 6, 8, 10, and 12. The SMS included diabetes education messages. Remote, individual, automated text messaging.
**Comparator**	Usual care (pre-crossover) Crossover study – see description of intervention section for further details.
**Outcomes of relevance to the review questions**	HbA1c at 3 months Only the 3-month pre-crossover outcome data were included, whereby cluster S was the intervention group and cluster P was the control.
**Commercial funding**	No
**Primary categories of self-management intervention according to PRISMS taxonomy**	1) Education about condition and management

BMI, body mass index; C, control group; CHW, community health worker; CKD, chronic kidney disease; DBP, diastolic blood pressure; DSME, diabetes self-management education; eGFR, estimated glomerular filtration rate; FBG, fasting blood glucose; GDM, gestational diabetes mellitus; Hb, hemoglobin; HbA1c, glycated hemoglobin; HDL, high-density lipoprotein; HRQoL, health-related quality of life; I, intervention group; LDL, low-density lipoprotein; NE, nutrition education group; NEP, nutrition education plus peer-support group; P, Centre Médical de Popenguine; PHC, primary health care center; PI, principle investigator; PRISMS, Practical Systematic Review of Self-Management Support; RCT, randomized controlled trial; S, Centre Philippe Maguilène Senghor; SBP, systolic blood pressure; SMBG, self-monitoring of blood glucose; T1DM, type 1 diabetes mellitus; T2DM, type 2 diabetes mellitus; TB, tuberculosis; TG, trigylcerides; TIA, transient ischemic attack; WC, waist circumference.

## Appendix V: Effect of self-management interventions on secondary outcome measures, including weight, body mass index, systolic blood pressure, diastolic blood pressure, total cholesterol, high-density lipoprotein, low-density lipoprotein, and triglyceride

David 2021^49^: Individually randomized, parallel-design RCT set in Nigeria, assessing a broad self-management education program compared with usual care.

Debussche 2018^18^: Individually randomized, parallel-design RCT set in Mali, assessing a peer-support intervention compared with usual care.

Farmer 2021^28^: Individually randomized, parallel-design RCT set in South Africa and Malawi, assessing an automated educational text-messaging-based intervention compared with a text-messaging sham intervention.

Gathu 2018^21^: Individually randomized, parallel-design RCT set in Kenya, assessing a broad self-management education program compared with usual care.

Muchiri 2016^23^: Individually randomized, parallel-design RCT set in South Africa, assessing a nutrition education intervention compared with enhanced usual care.

Muchiri 2021^48^: Individually randomized, parallel-design RCT set in South Africa, assessing a nutrition education intervention compared with enhanced usual care.

Oijeabu 2017^17^: Individually randomized, parallel-design RCT set in Nigeria, assessing a broad self-management education program compared with usual care.

Thuita 2020^24^: Individually randomized, parallel-design RCT with 3 arms set in Kenya; nutrition education with peer support compared with enhanced usual care included in these meta-analyses.

van Rooijen 2004^54^: Individually randomized, parallel-design RCT set in South Africa, assessing an exercise intervention with education compared with a relaxation-based sham intervention.

van Rooijen 2010^55^: Individually randomized, parallel-design RCT set in South Africa, assessing a broad self-management education program compared with usual care.

## Appendix VI: Subgroup and sensitivity analyses

David 2021^49^: Individually randomized, parallel-design RCT set in Nigeria, assessing a broad self-management education program compared to usual care.

Debussche 2018^18^: Individually randomized, parallel-design RCT set in Mali, assessing a peer-support intervention compared to usual care.

Gathu 2018^21^: Individually randomized, parallel-design RCT set in Kenya, assessing a broad self-management education program compared to usual care.

Muchiri 2016^23^: Individually randomized, parallel-design RCT set in South Africa, assessing a nutrition educxation intervention compared to enhanced usual care.

Muchiri 2021^48^: Individually randomized, parallel-design RCT set in South Africa, assessing a nutrition education intervention compared to enhanced usual care.

Ng’ang’a 2022^52^: Individually randomized, parallel-design RCT set in Rwanda, assessing a blood glucose self-monitoring intervention compared to usual care.

Thuita 2020^24^: Individually randomized, parallel-design RCT with 3 arms set in Kenya.
